# Fluctuations in Species-Level Protein Expression Occur during Element and Nutrient Cycling in the Subsurface

**DOI:** 10.1371/journal.pone.0057819

**Published:** 2013-03-05

**Authors:** Michael J. Wilkins, Kelly C. Wrighton, Carrie D. Nicora, Kenneth H. Williams, Lee Ann McCue, Kim M. Handley, Chris S. Miller, Ludovic Giloteaux, Alison P. Montgomery, Derek R. Lovley, Jillian F. Banfield, Philip E. Long, Mary S. Lipton

**Affiliations:** 1 Biological Sciences Division, Pacific Northwest National Laboratory, Richland, Washington, United States of America; 2 Department of Earth and Planetary Science, University of California, Berkeley, California, United States of America; 3 Earth Sciences Division, Lawrence Berkeley National Laboratory, Berkeley, California, United States of America; 4 Department of Microbiology, University of Massachusetts Amherst, Amherst, Massachusetts, United States of America; University of Illinois at Chicago, United States of America

## Abstract

While microbial activities in environmental systems play a key role in the utilization and cycling of essential elements and compounds, microbial activity and growth frequently fluctuates in response to environmental stimuli and perturbations. To investigate these fluctuations within a saturated aquifer system, we monitored a carbon-stimulated *in situ Geobacter* population while iron reduction was occurring, using 16S rRNA abundances and high-resolution tandem mass spectrometry proteome measurements. Following carbon amendment, 16S rRNA analysis of temporally separated samples revealed the rapid enrichment of *Geobacter*-like environmental strains with strong similarity to *G. bemidjiensis*. Tandem mass spectrometry proteomics measurements suggest high carbon flux through *Geobacter* respiratory pathways, and the synthesis of anapleurotic four carbon compounds from acetyl-CoA via pyruvate ferredoxin oxidoreductase activity. Across a 40-day period where Fe(III) reduction was occurring, fluctuations in protein expression reflected changes in anabolic versus catabolic reactions, with increased levels of biosynthesis occurring soon after acetate arrival in the aquifer. In addition, localized shifts in nutrient limitation were inferred based on expression of nitrogenase enzymes and phosphate uptake proteins. These temporal data offer the first example of differing microbial protein expression associated with changing geochemical conditions in a subsurface environment.

## Introduction

The activities of microbial populations play an important role in the cycling of metals and nutrients in environmental systems, where processes including element uptake, excretion and transformations catalyzed by microorganisms contribute to dynamic fluxes of C, P, N, S, and Fe [1]. Given that these fluxes are closely linked to the physiological state of microbial community members, the interrogation of *in situ* microbial metabolism and activity may offer an opportunity to better understand biogeochemical cycles.

Elucidating microbial metabolic pathways and activity in subsurface environments has traditionally been problematic, with microbial communities located in discrete pore spaces deep underground. Coupled to this, growth rates within complex, low biomass microbial communities are typically slow [2], [3], and governed by a range of factors including nutrient availability, limited concentrations of electron donors and acceptors, and other environmental stresses. However, the stimulation of *in situ* microbial activity via carbon amendment allows shifts in global protein and mRNA profiles to be measured under controlled conditions, where the enrichment of specific microbial groups can be predicted and subsequently monitored [4], [5].

This approach has been applied at the Rifle Integrated Field Research Challenge (IFRC) site in Western Colorado, where the use of *in situ* carbon amendment experiments over the past decade has allowed both microbiological and geochemical responses to be better predicted [6]. At this site, acetate amendment to the subsurface typically enriches Fe(III)-reducing *Geobacter* spp., both within the planktonic and sediment-associated communities [6]. Understanding the physiology and metabolism of *Geobacter* spp. in environmental systems is important for predicting biogeochemical processes and bioremediation efforts in the subsurface; these species and strains can couple the oxidation of organic carbon to the reduction of a range of metals including Fe, U, V, and Se 7,8,9,10. By accelerating the rate of these processes via carbon amendment, biogeochemical interactions can be investigated that would be extremely challenging to measure under background rates and conditions. These data then offer the potential to link metabolic and physiological inferences to geochemical measurements, and obtain a greater predictive understanding of subsurface processes.

At the Rifle IFRC site, acetate amendment to the subsurface stimulates Fe(III) reduction for approximately 30 days, during which *Geobacter* populations are thought to catalyze changes in Fe and U biogeochemistry [11]. Following this period, the development of sulfate-reducing conditions in the aquifer is linked to decreasing abundances and activity of planktonic *Geobacter* [11], [12]. To date, a number of approaches have been used to interrogate the physiology and ecology of *Geobacter* in the subsurface, including quantification of specific genes associated with N, P and acetate limitation [13], [14], [15]. During a previous carbon injection field experiment, shotgun proteomic analysis was used to investigate the whole expressed proteome of the stimulated planktonic *Geobacter* population [5]. This technique measures all the expressed proteins within a sample, and can be used to infer activity of specific microbial species. From this study, significant carbon flux through respiratory pathways of *Geobacter* species was inferred, and temporal strain-level shifts within the population were identified. However, due to the small number of proteomic samples recovered, temporal changes in the metabolism and physiology of the *Geobacter* population could not be accurately assessed. Data analysis suggested that the vast majority of *Geobacter* strains in the subsurface at the Rifle IFRC site were most closely related to *G. bemidjiensis*, a member of the “subsurface clade” of *Geobacter* [16].

We have expanded upon this previous work [17], recovering multiple planktonic biomass samples over a period of stimulated Fe(III) reduction in the subsurface during a subsequent carbon amendment experiment. Following the identification of a dominant *Geobacter* sub-population within these samples, shotgun proteomic analyses were used to track protein expression over a 40-day period in the subsurface at the Rifle IFRC. Using these data, we have linked shifting metabolism and physiology with measured geochemical parameters to better understand factors driving subsurface biogeochemical cycles including iron, nitrogen, carbon, and hydrogen transformations.

## Materials and Methods

### Injection Gallery Design & Operation

The field experiment was carried out during August and September 2010 (23^rd^ August –22^nd^ September) at the Rifle Integrated Field Research Challenge (IFRC) site, located approximately 200 miles west of Denver in Western Colorado (USA) (Co-ordinates +39° 31′ 45.60″, −107° 46′ 18.50″).

An injection gallery consisting of 10 injection wells, multiple down-gradient monitoring wells arranged in three rows, and three up-gradient monitoring wells was constructed using sonic rotary drilling (Figure S1). Acetate:bromide (50 mM:5 mM) amended groundwater was injected into the subsurface via the injection wells at approximately 16 L per injection well, per day. This rate resulted in a final groundwater concentration of ∼5 mM acetate, which served as a carbon source and electron donor over the course of the amendment experiment. Geochemical samples were taken from 5 m depth after purging 12 L groundwater from the sampling well. Ferrous iron and sulfide concentrations were analyzed immediately on site following sampling using the HACH 1,10 Phenanthroline and Methylene Blue colorimetric assays respectively (HACH, CO, USA). Acetate, bromide, and sulfate were also analyzed on site, using a Dionex ICS1000 ion chromatograph equipped with a CD25 conductivity detector and a Dionex IonPac AS22 column (Dionex, CA, USA). U(VI) values were determined using a Kinetic Phosphorescence Analyzer (KPA) (Chemchek, WA, USA). Additional details on geochemical analyses can be found in Williams et al [11].

### DNA and Protein Sample Collection

Nine biomass samples for proteomic analyses were recovered from groundwater over the course of the *in situ* biostimulation experiment, on the following days after the start of acetate injection: 5, 8, 10, 13, 15, 17, 29, 36, and 43. Samples for 16S rRNA analysis were recovered after 3, 8, 17, 24, and 29 days. All samples were recovered from well CD01, located 2 m down-gradient in the first row of down-gradient monitoring wells (Figure S1). For each sample, between 20–100 L of groundwater pumped at approximately 2 l min^−1^ from well CD01 was filtered through a pre-filter (1.2 µm, 293 mm diameter Supor disc filter, Pall Corporation, NY, USA), followed by 0.2 µm 293 mm diameter Supor disc filter. After filtration, filters were immediately frozen in an ethanol-dry ice mix, and shipped overnight on dry ice to Pacific Northwest National Laboratory for proteomic analysis.

### 16S rRNA Analysis

DNA was extracted from groundwater filters recovered 3, 8, 17, 24, and 29 days after the start of acetate amendment using the MoBio PowerMax Soil DNA extraction kit (Carlsbad, CA) and triplicate extracts for each sample were combined and concentrated using ethanol precipitation. Extracted DNA was used as a template for amplification of the 16S rRNA gene with the primers 27F (5′-AGAGTTTGATCCTGGCTCAG-3′) and 1492R (5′-GGTTACCTTGTTACGACTT-3′). To minimize PCR bias, amplicons from a gradient PCR reaction (20 cycles) were pooled and used as input for Illumina library preparation and HiSeq 2000 sequencing using standard protocols. EMIRGE analyses were performed as previously reported [18]. Briefly, for each sample (now a bar-coded library), quality-filtered trimmed reads were subsampled (1 million reads) at random without replacement. Each trimmed read subsample was input into an amplicon-optimized version of EMIRGE [18] for assembly into full-length genes. This code is freely available at https://github.com/csmiller/EMIRGE. EMIRGE was run for each subsample for 120 iterations with default parameters designed to merge reconstructed 16S rRNA genes ≥97% identical. Abundance estimates for each assembled 16S rRNA gene were derived by the probabilistic accounting in EMIRGE of how reads map to each assembled rRNA sequence [18]. The starting rRNA database was derived from version 102 of the SILVA SSU database, while taxonomy was assigned to each OTU using SILVA 108.

To examine the overall diversity and relative abundance in 16S rRNA sequences, results for organisms with relative abundance greater than 0.01% were included, with relative abundance data calculated by EMIRGE for the entire dataset. To demonstrate the relative abundance and phylogenetic affiliation of the most abundant *Geobacter spp.*, 16S rRNA sequences above 0.5% relative abundance were aligned using MUSCLE [19], and then incorporated into a neighbor-joining tree using MEGA [20]. Bootstrap analysis was carried out using 100 iterations. Relative abundance data was added to the phylogenetic tree using ITOL [21]. The 16S rRNA sequences used in this study are included in Table S1.

### Proteomic Sample Preparation

Rifle groundwater sample filters (0.2 µm) were removed from −80°C freezer individually for proteomics sample preparation as follows: frozen filters were crushed into small pieces and 6 g of the pieces were placed into a 50 mL Falcon tube. Lysis buffer (2% (w/v) SDS, 100 mM DTT in 100 mM ammonium bicarbonate pH 7.6, (Sigma-Aldrich, St. Louis, MO)) was added to the sample and vortexed and incubated at 95°C for 5 minutes. The tube was vortexed and spun to reduce the bubbles and the supernatant was added to 3 barocycle Pulse tubes (with rinsing of the filter pieces) and barocycled for 10 cycles (20 seconds at 35,000 psi back down to ambient pressure for 10 seconds) (Pressure Biosciences Inc., South Easton, MA). The sample was removed from the Pulse tubes and spun at 15,000×g for 5 minutes to pellet debris. The Filter-Aided Sample Preparation (FASP) [22] technique was used to remove the SDS from the sample. Two 15 mL 30 K MWCO spin filters (Millipore, Billerica, MA) were filled with 13 mL of 8 M urea in 100 mM ammonium bicarbonate, pH 8.5 (Sigma-Aldrich, St. Louis, MO) and 2 mL of sample in each. The spin filters were spun at 4000×g for 40 minutes to the dead volume of ∼200 µl and 10 mL of 8 M urea, pH 8.0 was added and spun again at 4000×g for 40 minutes. This step was repeated 3 times. Finally, 100 mM ammonium bicarbonate, pH 8.0 was added and spun at 4000×g for 40 minutes, with the step repeated once. A Coomassie Plus (Thermo Scientific, Rockford, IL) assay was used to determine protein concentration and 100 mM ammonium bicarbonate pH 8.0 was added to the sample to cover the filter. Tryptic digestion (Promega, Madison, WI) was performed at a 1∶50 (w/w) trypsin to protein ratio with the addition of 1 mM CaCl2 to stabilize the trypsin and reduce autolysis. The collection tube was cleaned and the sample was incubated overnight at 37°C. The following day the spin filter was spun at 4000×g for 30 minutes to collect the peptides. The filter was rinsed once with 100 mM ammonium bicarbonate, pH 8.0 and spun again at 4000×g for 30 minutes. The sample was cleaned via strong cation exchange (SCX) solid phase extraction (SPE) (Supelco, Bellefonte, PA) and dried in a speed-vac to 100µl and assayed with Bicinchoninic acid (BCA) (Thermo Scientific, Rockford, IL) to determine the final peptide concentration and vialed for 2D-LC-MS/MS analysis.

### 2D-LC-MS/MS Analysis

The 2D-LC system was custom built using two Agilent 1200 nanoflow pumps and one 1200 capillary pump (Agilent Technologies, Santa Clara, CA), various Valco valves (Valco Instruments Co., Houston, TX), and a PAL autosampler (Leap Technologies, Carrboro, NC). Full automation was made possible by custom software that allows for parallel event coordination providing near 100% MS duty cycle through use of two trapping and analytical columns. All columns were manufactured in-house by slurry packing media into fused silica (Polymicro Technologies Inc., Phoenix, AZ) using a 1 cm sol-gel frit for media retention. Column dimensions are as follows: first dimension SCX column; 5-µm PolySULFOETHYL A (PolyLC Inc., Columbia, MD), 15-cm ×360 µm outer diameter (o.d.) ×150 µm inner diameter (i.d.). Trapping columns; 5-µm Jupiter C_18_ (Phenomenex, Torrence, CA), 4-cm ×360 µm o.d. ×150 µm i.d. Second dimension reversed-phase columns; 3-µm Jupiter C_18_ (Phenomenex, Torrence, CA), 35-cm ×360 µm o.d. ×75 µm i.d. Mobile phases consisted of 0.1 mM NaH_2_PO_4_ (A) and 0.3 M NaH_2_PO_4_ (B) for the first dimension and 0.1% formic acid in water (A) and 0.1% formic acid in acetonitrile (B) for the second dimension.

MS analysis was performed using a LTQ Orbitrap Velos ETD mass spectrometer (Thermo Scientific, San Jose, CA) outfitted with a custom electrospray ionization (ESI) interface. Electrospray emitters were custom made using 150 µm o.d. ×20 µm i.d. chemically etched fused silica [23]. The heated capillary temperature and spray voltage were 275°C and 2.2 kV, respectively. Data were acquired for 100 min, beginning 65 min after sample injection and 15 min into gradient. Orbitrap spectra (AGC 1×10^6^) were collected from 400–2000 m/z at a resolution of 60 k followed by data dependent ion trap CID MS/MS (collision energy 35%, AGC 3×10^4^) of the ten most abundant ions. A dynamic exclusion time of 60 sec was used to discriminate against previously analyzed ions.

### Data Analysis

MS/MS data was searched using SEQUEST against a peptide database constructed from the *Geobacter bemidjiensis* genome, using relatively conservative filters [Xcorr values of 1.9 (+1), 2.2 (+2), and 3.5 (+3)]. Resulting peptide identifications were filtered using an MSGF cutoff value to 1 e−^10^ [24]. Peptides identified by only one spectral count were discarded. Spectral count data for each identified protein were normalized for protein length by dividing spectral counts by the amino acid protein length. These values were subsequently log transformed. These abundance values were converted to z-score values (also called the Standard Row Function). Z-scores were calculated by taking the mean protein abundance across all conditions, subtracting from this the individual protein abundance, and dividing this value by the standard deviation of the values. Where data was missing from one condition, the absent value was assigned a score equal to the lowest Z-score in the data matrix divided by 1.5. Likewise, the Z-scores for that protein in the other conditions were assigned a value equal to the highest Z-score in the data matrix multiplied by 1.5. This resulted in a presence/absence appearance in subsequent heat maps. Z-score values can be used to determine proteins showing significant changes from their average values. In this study, z-score values were considered significantly different if the difference was at least 2 or greater. Heat maps were generated using the TIGR software MeV (http://www.tm4.org/mev/). Probable orthologous proteins were identified using the protocol identified in Callister et al. [25], with version 4.1 of INPARANOID used in this study.

## Results and Discussion

**Figure 1:**
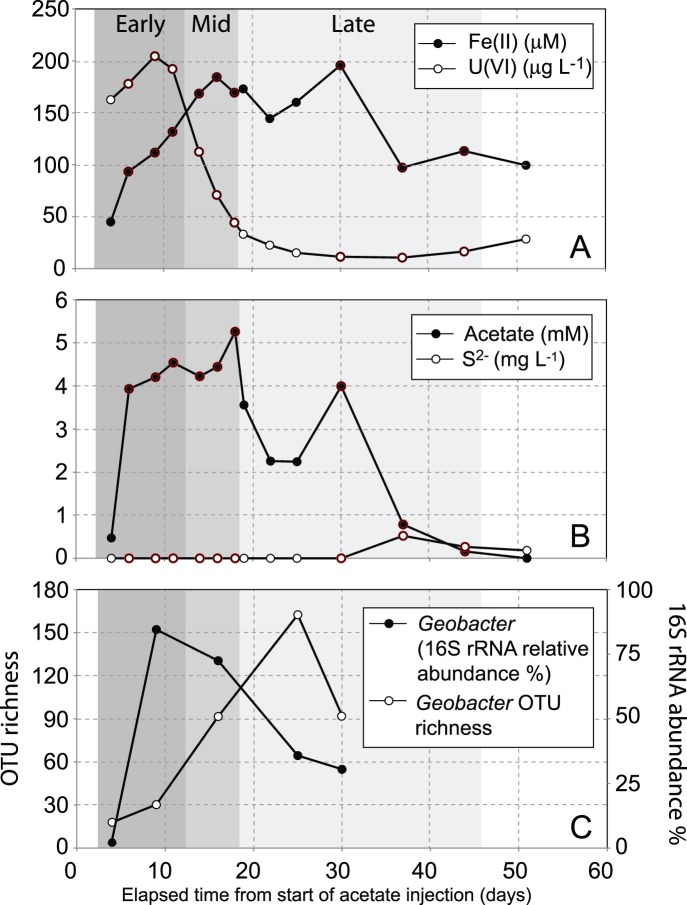
Geochemical and microbiological data obtained from downgradient well CD01 at the Rifle site, showing (A/B) the concurrent increase in Fe(II) and decrease in aqueous U(VI) associated with acetate arrival in the downstream monitoring well, and (C) the relative abundance of members of the *Geobacteraceae*, and *Geobacter* strain richness over time. Red circles around a data point indicate that a proteomic sample was collected.

Nine proteomic samples were collected from well CD-01 (located 2.5 m downgradient from the region of injection) and binned into three different phases of carbon amendment; Early (samples collected after 5, 8, and 10 days), Middle (samples collected after 13, 15, and 17 days), and Late (samples collected after 29, 36, and 43 days). (Figure 1A). Bins were assigned via hierarchical clustering of samples based on a suite of geochemical measurements (acetate, Fe(II), U(VI), sulfate, sulfide) taken at each time point (Figure S2). Acetate and bromide concentrations in groundwater were monitored in the sampling well to determine the start of biostimulation in that region of the aquifer. Increases in aqueous Fe(II) concentrations following the arrival of acetate in the first row of downgradient monitoring wells (Figure S1) (approximately 5 days after the start of injection) likely indicated the start of stimulated enzymatic Fe(III) reduction. Fe(II) values increased from background concentrations of ∼50 µM to between 100–150 µM during the early stages of the experiment. The middle stage of biostimulation was characterized by elevated Fe(II) concentrations (between 150–200 µM), before fluctuating and decreasing concentrations were monitored during the later stages of the experiment (Figure 1A). Concurrent to this, acetate concentrations followed similar trends (Figure 1B), while aqueous U(VI) concentrations increased slightly following the stimulation of microbial activity, and then decreased rapidly over a 10-day period to levels below the U.S. Environmental Protection Agency’s Maximum Contaminant Level (MCL) for uranium (Figure 1A). Finally, sulfide (S^2−^) concentrations were below detection for the first 30 days of the experiment, and only after 37 days were concentrations of aqueous S^2−^ measured (Figure 1B).

**Figure 2:**
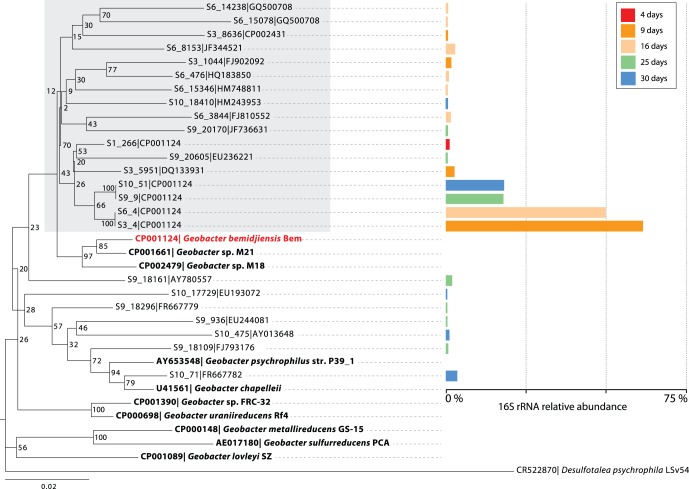
Neighbor-joining phylogenetic tree showing the placement of *Geobacter*-like environmental 16S rRNA sequences recovered from planktonic biomass at five time-points during carbon amendment. Bolded sequences show the placement of isolate *Geobacter* 16S rRNA sequences, in the context of the environmental sequences. Sequences within the grey box fall within the *G. bemidjiensis*/M21/M18 clade, and account for the majority of environmental *Geobacter* sequences recovered during this study. Accession numbers associated with the environmental sequences correspond to the best match when aligned to SILVA, Greengenes, and the RDP databases.

To confirm *Geobacter* dominance in the samples, as well as identify the temporal distribution of *Geobacter* strains, 16S rRNA gene sequences from 5 biomass samples (collected on days 3, 8, 17, 24, and 29) were analyzed. Results revealed that *Geobacter* strains were rapidly enriched within the microbial community following the arrival of acetate in the subsurface; the relative abundance of *Geobacter* 16S rRNA sequences increased from 2% to 85% over a 5 day period, before gradually decreasing over the remaining time points (Figure 1C). Complementary groundwater cell count data from an adjacent well confirmed that *Geobacter* cell numbers rapidly increased during the first ∼8 days of carbon amendment before leveling off [26]. Although *Geobacter* strain richness also increased over time, only a few strains were responsible for the majority of *Geobacter* dominance over the course of the experiment. This observation suggests that a small number of fast-growing *Geobacter* strains responded to the presence of acetate, and were subsequently complemented by strains that either exhibited slower growth rates, or were able to occupy specific biogeochemical niches during the later period of carbon amendment (Figures 1C and 2). This finding is supported by previous studies demonstrating that *Geobacter* strains were significantly enriched following acetate amendment at the Rifle IFRC site 6,11,27. Within the *Geobacteraceae,* phylogenetic placement of the recovered 16S rRNA sequences revealed that indigenous *Geobacter* strains closely related to *G. bemidjiensis* were the dominant members of this population (Figure 2, Table S2). Other more distantly related strains emerged in later time points, but contributed a much smaller relative fraction of 16S rRNA sequences (<5%) (Figure 2).

To investigate how dominant *Geobacter* strains responded to excess carbon flux into the local environment, planktonic biomass was sampled at nine time points and analyzed using high-resolution proteomic 2D-LC-MS/MS measurements. In instances such as this, where metagenomic sequence data is unavailable, genomic information from sequenced strains closely-related to environmental species can be used to search mass spectrometry data; conserved protein sequences between closely-related strains allow predicted peptides (from a sequenced isolate) to be matched to measured mass spectra (from an environmental sample) [17], [28]. This study was aided by the availability of genomic information from multiple sequenced *Geobacter* isolates, including *Geobacter* species strains M18 and M21, and *G. uraniireducens* that were all isolated from the Rifle site. Within the *Geobacter*, a fraction of proteins encoded by these isolate genomes are conserved; patterns of orthologous proteins were assessed, and used to identify 1116 orthologous proteins across all eight genomes that represented a *Geobacter* “core” proteome that would likely be present in environmental strains (Table S3). While this number represented a significant fraction of protein coding genes within each organism (Table 1), the number of orthologs was even higher between a few closely related *Geobacter* strains; *G. bemidjiensis* and the Rifle site isolate strain M21 share 2561 orthologous proteins, with 94% amino acid similarity across these orthologs [17]. Given (1) the phylogenetic similarity of the majority of dominant environmental strains to the *G. bemidjiensis*/M18/M21 clade (grey box in Figure 2), (2) the high number of orthologs shared between *G. bemidjiensis*, M18 and M21, (3) the desire to limit redundancy with the search database, and (4) the well annotated and curated nature of the *G. bemidjiensis* genome, predicted peptides from the *G. bemidjiensis* genome were used to search the proteomic MS/MS data.

**Table 1:** Number of orthologous proteins across eight *Geobacter* genomes that comprise a “core” proteome.

	Gbem	GM21	GM18	Gura	GFRC32	Gsul	Gmet	Glov
Protein-coding genes	4034	4152	4523	4430	3839	3465	3576	3725
Fraction of orthologous genes commonto all *Geobacter* strains	28%	27%	25%	25%	29%	32%	31%	30%
Expressed fraction	13%	13%	12%	12%	14%	15%	15%	14%

The expressed fraction refers to *Geobacter bemidjiensis* proteins detected within this data set, extrapolated across the additional seven *Geobacter* genomes.

**Figure 3:**
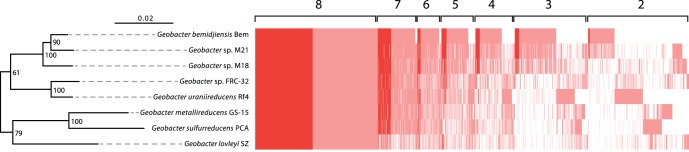
Distribution and expression of orthologous proteins across eight *Geobacter* genomes. Pink shading illustrates the presence of orthologous proteins within genomic data, while red shading indicates expression of an orthologous protein by an environmental *Geobacter* strain, identified using predicted peptides from *G. bemidjiensis*. Values along the top of the chart indicate the number of *Geobacter* strains the orthologs are distributed over. For the core proteome (as identified by orthologs present in all eight *Geobacter* genomes), ∼47% expression is detected within biomass recovered from the Rifle subsurface. The data is coupled to a neighbor-joining tree constructed using 16S rRNA sequences from eight sequenced *Geobacter* genomes, and illustrates the correlation between inferred evolutionary distance and the distribution of orthologous proteins.

Across nine planktonic biomass samples, over 900 proteins from environmental *Geobacter* strains that match the *G. bemidjiensis* proteome were subsequently detected (Table S4). Despite the challenges associated with measuring peptides from environmental samples, 530 of the 1116 proteins (∼47%) comprising a “core” *Geobacter* proteome were detected in all nine samples. These results confirm (1) the ability to identify and detect a significant number of conserved proteins from environmental strains using closely-related isolate genomic data in search databases, and (2), that the activity of the identified core enzymes extends to maintaining growth and survival of environmental strains in subsurface environments (Table S4). In total, 718 of ∼900 *G. bemidjiensis* proteins detected within these samples have orthologs in at least one other *Geobacter* strain (Figure 3), with the highest number shared between *G. bemidjiensis* and strain M21 (712 detected orthologs).

### Temporal Geochemical-Proteomic Analyses

**Figure 4:**
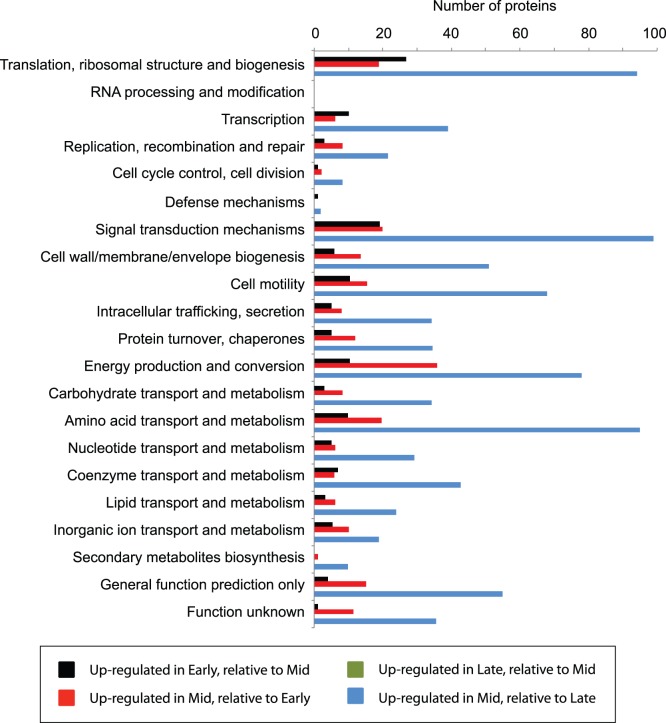
Significant shifts in protein abundances between the three stages of carbon amendment, binned into COG categories. Significant protein abundance increases and decreases between the stages were inferred using Z-score calculations.

Significant protein abundance shifts were investigated across the three different geochemical stages (early, middle, late) of biostimulation (Figure S2). Overall trends were indicative of a population responding to stimulation (such as the sudden availability of carbon), and were similar to growth patterns measured for *Geobacter* strains within laboratory settings in batch cultures [9]; initial rapid growth of the Geobacter population was inferred by statistically significant (P<0.05) abundance increases (relative to later stages of biostimulation) for proteins associated with biogenesis (Figure 4, Table S5). As an example, 34% of the detected ribosomal proteins (**C**luster of **O**rthologous **G**ene (COG) category J) were at greater abundances in samples recovered during the early stage of biostimulation, compared to 12% in the middle stage. This carbon usage results in a *Geobacter* biomass “bloom” within biostimulated regions of the aquifer, as inferred by 16S rRNA relative abundances (Figure 2), and cell count data [26]. In addition, similar observations have been reported in earlier carbon amendment experiments in the Rifle subsurface [6], [11]. During the middle stage of biostimulation, slowing of *Geobacter* growth was inferred from decreasing abundances of proteins associated with biogenesis (as described above) (Figure 4). Conversely however, abundance increases (P<0.05) were observed in proteins associated with energy generation (COG category C) and amino acid metabolism and transport (COG category E) (Figure 4) over the same time period, consistent with some level of increasing respiration and cell maintenance. Finally, measured abundances decreased for large numbers of proteins between the subsequent middle and late stages, indicating that significant losses in growth and activity occur in the planktonic *Geobacter* population over this time period.

It is worth noting that the shifts in protein abundances reported here do not simply correspond to changes in organism abundances, as displayed in Figure 2. The fraction of *Geobacter* 16S rRNA sequences as a total of the whole microbial population decreases between the early and middle stages of carbon amendment (Figure 1C), and yet increases are observed in certain protein abundances over this same time period. These changes within specific pathways are presented below, and reveal physiological shifts occurring over the period of carbon amendment within the Rifle aquifer.

### Acetate Activation and Utilization

**Figure 5:**
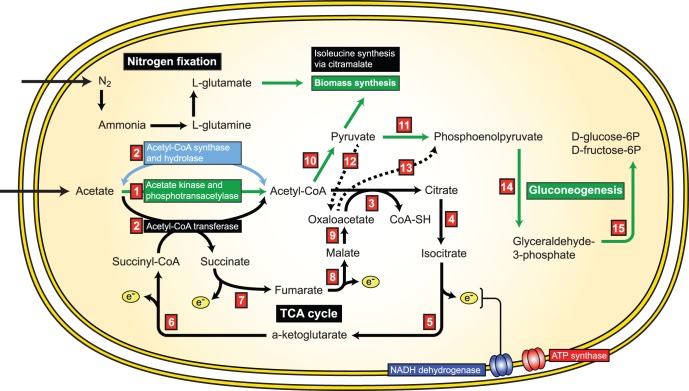
Central metabolism in indigenous *Geobacter* strains as inferred from proteomic data. Red number-containing boxes refer to specific enzymes in figure 6. Adapted from Mahadevan et al. [30].

A key characteristic of *Geobacter* strains is their efficient uptake and use of acetate. This carbon compound is utilized via two different pathways, both of which activate acetate to acetyl-CoA. The first pathway involves the enzyme acetyl-CoA transferase (ATO) (Gbem_0468, Gbem_0795), which has two functions in *Geobacter* strains: the activation of acetate to acetyl-CoA, and the conversion of succinyl-CoA to succinate as part of the tricarboxylic acid (TCA) cycle [29]. Because of this coupling (Figure 5), acetyl-CoA produced via this mechanism can be completely consumed via condensation with citrate to form oxaloacetate (Figure 5). Additional acetyl-CoA must therefore be synthesized for biosynthetic reactions via a two-step reaction involving acetate kinase (ACK) and phosphotransacetylase (PTA). This acetyl-CoA is then converted to pyruvate via a pyruvate ferredoxin oxidoreductase (PFOR) operating in reverse (Figure 5) [30].

**Figure 6:**
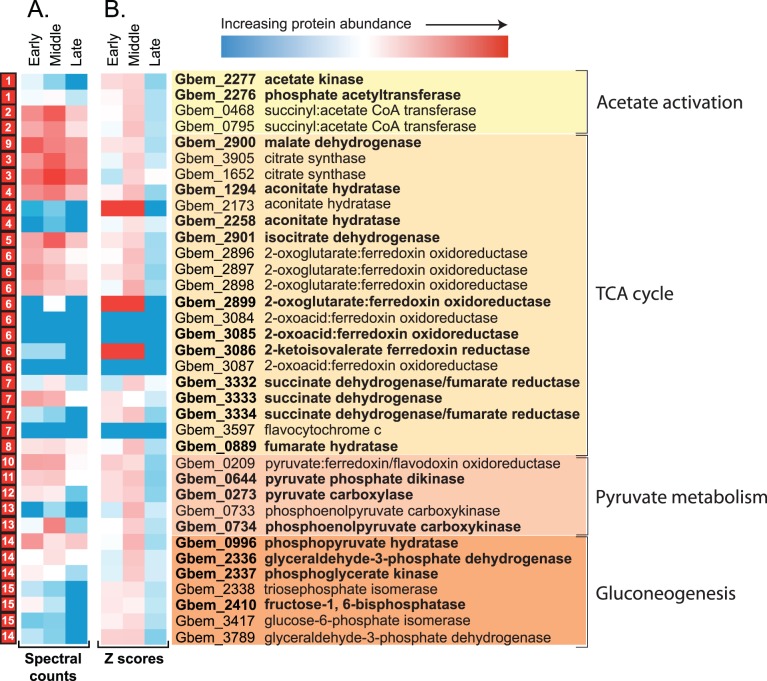
Relative abundance data for central metabolic pathways outlined in **figure 4**, using both log transformed spectral count information (A), and Z-scores (B) to better identify relative abundance shifts across the three stages of carbon amendment. Proteins that are orthologous across all eight sequenced *Geobacter* species are highlighted bold.

There is proteomic support for both acetate-activation pathways throughout the datasets, with ATO pathway components (Gbem_0468, Gbem_0795) present at greater abundances than ACK (Gbem_2277) and PTA (Gbem_2276) across all phases of carbon amendment (Figure 6A). Significant shifts in protein abundances (P<0.05) between the sample stages were inferred using Z score calculations (Figure 6B) [31], and revealed changing trends in carbon utilization. Both ATO enzymes (Gbem_0795, Gbem_0468) increased in abundance between the early and middle stages of biostimulation, indicative of increasing flux through respiratory pathways. Further emphasizing the importance of energy generation, many TCA cycle enzymes were highly abundant across all three sample stages, with citrate synthase (Gbem_3905, Gbem_1652), isocitrate dehydrogenase (Gbem_2901), aconitate hydratase (Gbem_1294), and succinate dehydrogenase (Gbem_3332) all increasing in abundance from the early to middle period of biostimulation (Figure 6B). Indeed, these three enzymes contribute to the ∼17% of proteins showing abundance increases across this period that are associated with energy generation and conversion (COG category C) (Figure 4). Inferred high fluxes of carbon through respiratory pathways are supported by *in silico* predictions for the closely related species *Geobacter sulfurreducens.* Data from the in silico study suggests that >90% of consumed acetate is directed to the TCA cycle for respiration when growing on Fe(III) [32].

Mirroring trends identified within the COG classification data (Figure 4), proteomic data suggests that while energy generation was presumably increasing over this time period, carbon flux to biosynthesis was not concurrently up regulated. Neither ACK nor PTA enzymes showed significant abundance increases between the early and middle stages of the experiment. A similar trend was observed for the PFOR enzyme (Gbem_0209), that converts acetyl-CoA to pyruvate (Figure 6B). The activity of this enzyme is the primary mechanism for generating 4-carbon compounds that are necessary for growth when acetate is the primary carbon source [30]. From these and other protein abundances associated with central metabolism, we can infer that (1) the flux passing through respiratory pathways may increase during the middle state of biostimulation, and (2) consequently, a larger fraction of carbon flux occurs towards biosynthesis during the early period of the experiment relative to later stages, indicative of *Geobacter* cell growth and proliferation following the initial arrival of carbon in the subsurface. However, it is worth noting that pyruvate carboxylase (Gbem_0273) can channel pyruvate synthesized via PFOR into respiratory pathways (via conversion to oxaloacetate). Given that this enzyme was detected within the proteomic results, the flux of carbon towards respiratory pathways may be even greater than is reflected within these data.

The identification of potential shifts in carbon flux through central metabolism has implications for metal biogeochemical cycles and bioremediation. Our results hint at complex linkages between cellular metabolism and the extracellular environment. Here proteomic inferences suggest a larger fraction of carbon was shunted to respiratory reactions rather than anapleurotic reactions during the middle stage of carbon amendment, when U(VI) was effectively removed from solution (Figure 1A). While these results may indicate that these metabolic shifts play a direct role in the efficiency of enzymatic U(VI) reduction, we note the lag in U(VI) reduction may also be indirectly impacted by biostimulation activities. Specifically, higher amounts of reactive Fe(III) present in early biostimulation could abiotically re-oxidize U(IV) phases in the aquifer, thereby masking active U(VI) reduction [33]. As carbon amendment progresses, the disappearance of more reactive Fe(III) phases (due to biological enzymatic dissolution) and increased concentrations of U(IV) (potentially due to shift in central metabolism from biosynthesis to respiration) may dilute these U(IV) reoxidation effects.

### Alternative Electron Donors

While these data suggest that a significant fraction of carbon flux is directed towards respiration in subsurface *Geobacter* strains, uptake hydrogenases may also play a role in driving respiratory processes in the Rifle aquifer. *Geobacter bemidjiensis* contains multiple genes encoding uptake hydrogenases [34], potentially expanding the range of electron donors that can be utilized for respiration. Both small and large subunits of NiFe hydrogenases (Gbem_3139, Gbem_3136, Gbem_3884) were detected within the proteomic samples, and as with other enzymes associated with energy generation, increases in hydrogenase abundance were observed between the early and middle sample stages (supplementary information). These hydrogenases therefore presumably contribute to increased rates of respiratory processes that were already inferred from protein abundances. The potential for hydrogenase activity within this population is perhaps unexpected; given the relatively high concentrations of aqueous carbon that can be utilized as an electron donor, the additional utilization of hydrogen for respiration may not be essential for survival. However, hydrogenase expression in this instance may be an example of this population maximizing energy generation during exposure to relatively carbon rich environmental conditions. In addition, recent studies have suggested a role for hydrogenases as part of the oxidative stress response in *Geobacter* species [35]. A function in oxidative stress would correlate to the central metabolism carbon flux profiles we infer here, when there is a shift from anabolic to respiratory processes, thus increasing oxidative stress. The increased abundance of a manganese and iron superoxide dismutase (Gbem_2204) over this time period may reflect another response to this stress.

### Nutrient Limitation

During the middle and late stages of Fe(III) reduction, any increase in the ratio of respiration/biosynthesis may reflect a slowing growth rate and could be associated with limiting nutrient concentrations that limit biomass production. As biomass is synthesized within the aquifer, essential nutrients and elements may become growth limiting. *Geobacter* utilize a number of common strategies for coping under these conditions, including fixing atmospheric N_2_ via nitrogenase activity [36], and expressing P uptake mechanisms [14]. However, no clear patterns of nitrogenase expression were identified in this data set. Although nitrogenase enzymes (NifK, NifD, NifH) were detected across all three stages of biostimulation (Table S4), previous measurements of bulk aqueous ammonium concentrations from nearby wells in the Rifle aquifer have suggested that non-limiting N concentrations are present during carbon amendment [13]. However, given the heterogeneous nature of the subsurface at Rifle [37], nitrogenase expression may reflect the development of local N-limiting regions within the aquifer around high biological activity. If limiting N concentrations are present in the subsurface during carbon amendment, the ability to fix nitrogen potentially offers *Geobacter* species a competitive advantage over other subsurface bacterial strains that are unable to carry out this process.

Given the low P concentrations within Rifle groundwater [14], indigenous *Geobacter* strains express high-affinity phosphate ABC transporters for P uptake. Both ATPase subunits and periplasmic binding proteins encoded by the *pst*-*pho* operon were observed within the dataset, while a phosphate selective porin (Gbem_4031) increased in abundance from the early to middle phase of carbon amendment. Interestingly, one phosphate ABC transporter increased in abundance over this same time period (Gbem_1847), while another decreased in abundance (Gbem_1710). Given that both transporters are associated with the high-affinity *pst* system, these differing expression patterns suggest that they may occupy different physiological roles in the subsurface. Ultimately however, the expression of components of the *pst-pho* operon across all stages of carbon amendment indicates that phosphate limitation is likely a key process affecting biostimulated microorganisms.

### Conclusions

Proteomic investigations had previously focused on acetate-stimulated planktonic biomass at the Rifle IFRC [5]. While these results had identified potential strain level shifts within the microbial community, and allowed central metabolism to be studied, the lack of a temporal series of samples had precluded statistical analyses of shifts in protein expression over the duration of biostimulation. In this study, we have utilized a greater number of samples to investigate the *in situ* temporal response of a microbial population to increased carbon availability during a biostimulation experiment. *Geobacter* strains were rapidly enriched within the planktonic microbial community upon acetate amendment and likely contributed to rapidly increasing aqueous Fe(II) concentrations over the first 15 days of the experiment. Physiological inferences suggest that the “bloom” of *Geobacter* biomass within the aquifer was associated with the efficient utilization of acetate for both respiration and biosynthesis, with potential shifts in carbon flux through anabolic and catabolic reactions over time. These temporal physiological changes have direct impacts on the aquifer biogeochemistry; the potential for increasing flux through respiratory pathways at certain time points has significant implications for elemental cycling in subsurface environments; electrons are thought to be primarily transferred to oxidized iron minerals, liberating soluble Fe(II) and any other adsorbed compounds into groundwater. However, these strains have the potential to dump electrons onto a wide range of redox-active metals and compounds, including organic matter (humic compounds), vanadium, and uranium, and therefore alter their physical and chemical behavior. Concurrently, decreasing biosynthesis in *Geobacter* strains may be linked indirectly to increasing activity of sulfate-reducing bacteria (SRB), as has been reported previously [12], [38]. Greater activity of SRB results in rising aqueous sulfide concentrations which can subsequently react with other metal cations to form precipitates and clog pore networks, catalyze the dissolution of Fe(III) phases, and release adsorbed metal cations from Fe(III) mineral surfaces. These data emphasize the tight biogeochemical linkages that exist between microbial assemblages and the surrounding local environment, and the metabolic shifts that occur within a population in response to these environmental stimuli.

## Supporting Information

Figure S1 **Plot layout at the Rifle IFRC.** Acetate injection wells are labeled CG-01 thru CG-10. Downgradient monitoring well CD-01 is highlighted with a red box.
(TIFF)

Figure S2 **Proteomic sample clustering for quantitative analysis.** Samples collected after 5, 8, and 10 days were grouped into the “early” phase of biostimulation, 13, 15, and 17 days into the “middle” stage, and 29, 36, and 43 days into the “late” stage. Clustering was performed using geochemical data (square root transformed) from each sampling time point (Fe(II), S^2−^, U, Acetate, and Sulfate) in R using the dist and hclust functions. Euclidean distances were calculated with average linkages between samples.
(EPS)

Table S1 **Environmental 16S rRNA sequences used during phylogenetic tree construction in this study.**
(DOCX)

Table S2 **Nucleotide % similarity between full length 16S rRNA sequences from environmental and sequenced strains.**
(DOCX)

Table S3 **Predicated orthologous proteins across eight sequenced** ***Geobacter*** **genomes.**
(XLSX)

Table S4 **Shotgun proteomic data, showing raw spectral counts, normalized spectral counts, and calculated Z scores across the nine samples.**
(XLSX)

Table S5 **Proteins exhibiting significant changes in abundance between the three stages of biostimulation, displayed as a percentage of the total number of proteins detected across the experiment (925).**
(DOCX)

## References

[1] Konopka A Ecology, Microbial. In: Schaechter M, editor. Encyclopedia of Microbiology. Oxford: Elsevier. 91–106.

[2] Lin B, Westerhoff HV, Röling WFM (2009) How *Geobacteraceae* may dominate subsurface biodegradation: physiology of *Geobacter metallireducens* in slow-growth habitat-simulating retentostats. Enviro Microbiol 11: 2425–2433. 10.1111/j.1462-2920.2009.01971.x 19638178

[3] Mailloux BJ, Fuller ME (2003) Determination of In Situ Bacterial Growth Rates in Aquifers and Aquifer Sediments. Appl Environ Microbiol 69: 3798–3808. 12839747 10.1128/AEM.69.7.3798-3808.2003 PMC165164

[4] Holmes DE, O’Neil RA, Chavan MA, N’Guessan LA, Vrionis HA, et al (2008) Transcriptome of *Geobacter uraniireducens* growing in uranium-contaminated subsurface sediments. ISME J 3: 216–230. 18843300 10.1038/ismej.2008.89

[5] Wilkins MJ, Verberkmoes NC, Williams KH, Callister SJ, Mouser PJ, et al (2009) Proteogenomic monitoring of *Geobacter* physiology during stimulated uranium bioremediation. Appl Environ Microbiol 75: 6591–6599. 19717633 10.1128/AEM.01064-09 PMC2765142

[6] Anderson RT, Vrionis HA, Ortiz-Bernad I, Resch CT, Long PE, et al (2003) Stimulating the In Situ Activity of *Geobacter* Species To Remove Uranium from the Groundwater of a Uranium-Contaminated Aquifer. Appl Environ Microbiol 69: 5884–5891. 14532040 10.1128/AEM.69.10.5884-5891.2003 PMC201226

[7] Lovley DR, Phillips ER (1988) Novel mode of microbial energy metabolism: organic carbon oxidation coupled to dissimilatory reduction of iron or manganese. Appl Environ Microbiol 54: 1472–1480. 16347658 10.1128/aem.54.6.1472-1480.1988 PMC202682

[8] Lovley DR, Phillips EJP, Gorby YA, Landa E (1991) Microbial reduction of uranium. Nature 350: 413–416.

[9] Ortiz-Bernad I, Anderson RT, Vrionis HA, Lovley DR (2004) Vanadium Respiration by *Geobacter metallireducens*: Novel Strategy for In Situ Removal of Vanadium from Groundwater. Appl Environ Microbiol 70: 3091–3095. 15128571 10.1128/AEM.70.5.3091-3095.2004 PMC404428

[10] Pearce CI, Pattrick RAD, Law N, Charnock JC, Coker VS, et al (2009) Investigating different mechanisms for biogenic selenite transformations: *Geobacter sulfurreducens, Shewanella oneidensis and Veillonella atypica* . Environ Technol 30: 1313–1326. 19950474 10.1080/09593330902984751

[11] Williams KH, Long PE, Davis JA, Wilkins MJ, N’Guessan AL, et al (2011) Acetate availability and its influence on sustainable bioremediation of uranium-contaminated groundwater. Geomicro J 28: 519–539.

[12] Barlett M, Zhuang K, Mahadevan R, Lovley DR (2011) Integrative analysis of the interactions between *Geobacter* spp. and sulfate-reducing bacteria during uranium bioremediation. Biogeosciences Discuss 8: 11337–11357.

[13] Mouser PJ, N’Guessan AL, Elifantz H, Holmes DE, Williams KH, et al (2009) Influence of heterogeneous ammonium availability on bacterial community structure and the expression of nitrogen fixation and ammonium transporter genes during in situ bioremediation of uranium-contaminated groundwater. Environ Sci Technol 43: 4386–4392. 19603651 10.1021/es8031055

[14] N’Guessan AL, Elifantz H, Nevin KP, Mouser PJ, Methe B, et al (2010) Molecular analysis of phosphate limitation in *Geobacteraceae* during the bioremediation of a uranium-contaminated aquifer. ISME J 4: 253–266. 20010635 10.1038/ismej.2009.115

[15] Elifantz H, N’Guessan LA, Mouser PJ, Williams KH, Wilkins MJ, et al (2010) Expression of acetate permease-like (apl) genes in subsurface communities of *Geobacter* species under fluctuating acetate concentrations. FEMS Microbiol Ecol 73: 441–449. 20533942 10.1111/j.1574-6941.2010.00907.x

[16] Holmes DE, O’Neil RA, Vrionis HA, N’Guessan LA, Ortiz-Bernad I, et al (2007) Subsurface clade of *Geobacteraceae* that predominates in a diversity of Fe(III)-reducing subsurface environments. ISME J 1: 663–677. 18059491 10.1038/ismej.2007.85

[17] Wilkins MJ, Callister SJ, Miletto M, Williams KH, Nicora CD, et al (2010) Development of a biomarker for *Geobacter* activity and strain composition; Proteogenomic analysis of the citrate synthase protein during bioremediation of U(VI). Microb Biotech 4: 55–63. 10.1111/j.1751-7915.2010.00194.x PMC3815795 21255372

[18] Miller CS, Handley KM, Wrighton KC, Frischkorn KR, Thomas BC, et al.. (2013) Short-read assembly of full-length 16S amplicons reveals bacterial diversity in subsurface sediments. PLoS ONE In Press. 10.1371/journal.pone.0056018 PMC3566076 23405248

[19] Edgar RC (2004) MUSCLE: multiple sequence alignment with high accuracy and high throughput. Nucleic Acids Res 32: 1792–1797. 15034147 10.1093/nar/gkh340 PMC390337

[20] Tamura K, Peterson D, Peterson N, Stecher G, Nei M, et al (2011) MEGA5: Molecular Evolutionary Genetics Analysis Using Maximum Likelihood, Evolutionary Distance, and Maximum Parsimony Methods. Mol Biol Evol 28: 2731–2739. 21546353 10.1093/molbev/msr121 PMC3203626

[21] Letunic I, Bork P (2011) Interactive Tree Of Life v2: online annotation and display of phylogenetic trees made easy. Nucleic Acids Res 39: W475–W478. 21470960 10.1093/nar/gkr201 PMC3125724

[22] Wisniewski JR, Zougman A, Nagaraj N, Mann M (2009) Universal sample preparation method for proteome analysis. Nat Meth 6: 359–362. 10.1038/nmeth.1322 19377485

[23] Kelly RT, Page JS, Luo Q, Moore RJ, Orton DJ, et al (2006) Chemically Etched Open Tubular and Monolithic Emitters for Nanoelectrospray Ionization Mass Spectrometry. Anal Chem 78: 7796–7801. 17105173 10.1021/ac061133r PMC1769309

[24] Kim S, Gupta N, Pevzner PA (2008) Spectral Probabilities and Generating Functions of Tandem Mass Spectra: A Strike against Decoy Databases. J Prot Res 7: 3354–3363. 10.1021/pr8001244 PMC2689316 18597511

[25] Callister SJ, McCue LA, Turse JE, Monroe ME, Auberry KJ, et al (2008) Comparative Bacterial Proteomics: Analysis of the Core Genome Concept. PLoS ONE 3: e1542. 18253490 10.1371/journal.pone.0001542 PMC2213561

[26] Holmes DE, Giloteaux L, Barlett M, Chavan MA, Smith JA, et al. (2013) Molecular Analysis of the In Situ Growth Rate of Subsurface *Geobacter* Species. Appl Environ Microbiol In Press. 10.1128/AEM.03263-12 PMC3591973 23275510

[27] Vrionis HA, Anderson RT, Ortiz-Bernad I, O’Neill KR, Resch CT, et al (2005) Microbiological and geochemical heterogeneity in an in situ uranium bioremediation field site. Appl Environ Microbiol 71: 6308–6318. 16204552 10.1128/AEM.71.10.6308-6318.2005 PMC1265972

[28] Denef VJ, Shah MB, VerBerkmoes NC, Hettich RL, Banfield JF (2007) Implications of strain- and species-level sequence divergence for community and isolate shotgun proteomic analysis. J Prot Res 6: 3152–3161. 10.1021/pr0701005 17602579

[29] Segura D, Mahadevan R, Juarez K, Lovley DR (2008) Computational and experimental analysis of redundancy in the central metabolism of *Geobacter sulfurreducens*. PloS Comput Biol 4. 10.1371/journal.pcbi.0040036 PMC2233667 18266464

[30] Mahadevan R, Palsson Bò, Lovley DR (2011) In situ to in silico and back: elucidating the physiology and ecology of *Geobacter* spp. using genome-scale modelling. Nat Rev Micro 9: 39–50. 10.1038/nrmicro2456 21132020

[31] Ding YHR, Hixson KK, Aklujkar MA, Lipton MS, Smith RD, et al (2008) Proteome of *Geobacter sulfurreducens* grown with Fe(III) oxide or Fe(III) citrate as the electron donor. Biochim Biophys Acta 1784: 1935–1941. 18638577 10.1016/j.bbapap.2008.06.011

[32] Tang YJJ, Chakraborty R, Martin HG, Chu J, Hazen TC, et al (2007) Flux analysis of central metabolic pathways in *Geobacter metallireducens* during reduction of soluble Fe(III)-nitrilotriacetic acid. Appl Environ Microbiol 73: 3859–3864. 17468285 10.1128/AEM.02986-06 PMC1932749

[33] Finneran KT, Anderson RT, Nevin KP, Lovley DR (2002) Potential for bioremediation of uranium-contaminated aquifers with microbial U(VI) reduction. Soil Sed Contam 11: 339–357.

[34] Coppi MV (2005) The hydrogenases of *Geobacter sulfurreducens*: a comparative genomic perspective. Microbiol 151: 1239–1254. 10.1099/mic.0.27535-0 15817791

[35] Tremblay P-L, Lovley DR (2012) Role of the NiFe hydrogenase Hya in oxidative stress defense in *Geobacter sulfurreducens* . J Bact 194: 2248–2253. 22366414 10.1128/JB.00044-12 PMC3347080

[36] Bazylinski DA, Dean AJ, Schuler D, Phillips EJP, Lovley DR (2000) N-2-dependent growth and nitrogenase activity in the metal-metabolizing bacteria, *Geobacter* and *Magnetospirillum* species. Environ Microbiol 2: 266–273. 11200427 10.1046/j.1462-2920.2000.00096.x

[37] Campbell KM, Kukkadapu RK, Qafoku NP, Peacock AD, Lesher E, et al (2012) Geochemical, mineralogical and microbiological characteristics of sediment from a naturally reduced zone in a uranium-contaminated aquifer. Appl Geochem 27: 1499–1511.

[38] Druhan JL, Steefel CI, Molins S, Williams KH, Conrad ME, et al (2012) Timing the Onset of Sulfate Reduction over Multiple Subsurface Acetate Amendments by Measurement and Modeling of Sulfur Isotope Fractionation. Environ Sci Technol 46: 8895–8902. 22834766 10.1021/es302016p

