# Supplementary figures and images for: Fluctuations in Species-Level Protein Expression Occur during Element and Nutrient Cycling in the Subsurface
Source: PLoS One. 2013 Mar 5;8(3):e57819. doi: 10.1371/journal.pone.0057819 (PMC3589452; doi:10.1371/journal.pone.0057819)

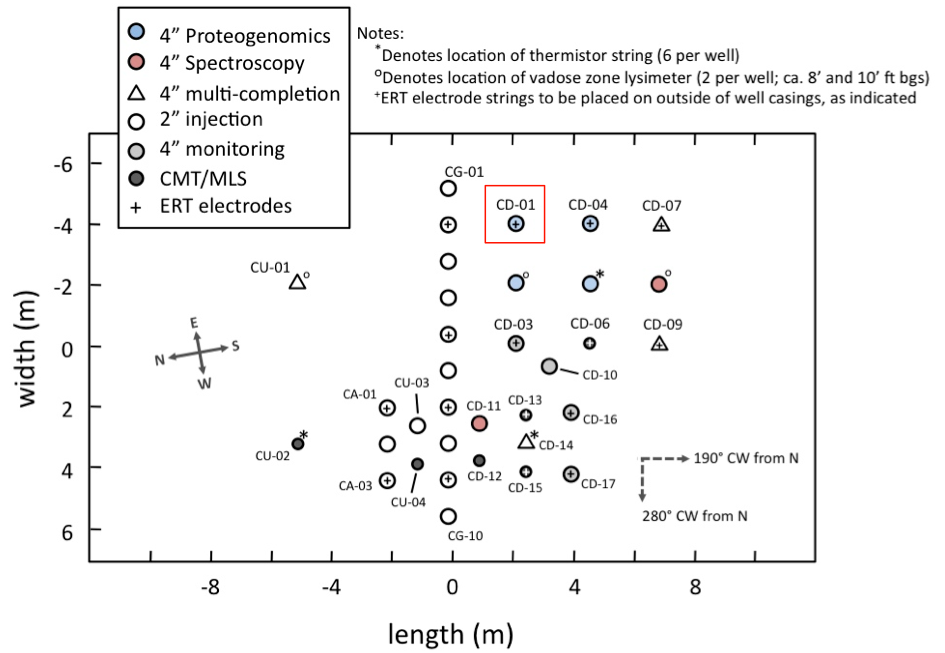

Supplement: Figure S1 — Plot layout at the Rifle IFRC. Acetate injection wells are labeled CG-01 thru CG-10. Downgradient monitoring well CD-01 is highlighted with a red box. (TIFF) [file pone.0057819.s001.tiff]
