# Supplementary material for: Fluctuations in Species-Level Protein Expression Occur during Element and Nutrient Cycling in the Subsurface
Source: PLoS One. 2013 Mar 5;8(3):e57819. doi: 10.1371/journal.pone.0057819 (PMC3589452; doi:10.1371/journal.pone.0057819)
Supplement: Table S1 — Environmental 16S rRNA sequences used during phylogenetic tree construction in this study. (DOCX) [file pone.0057819.s003.docx]

Environmental 16S rRNA sequences used in tree construction:

>S1_266|CP001124

AAGAGTTTGATCCTGGCTCAGAACGAACGCTGGCGGCGTGCTTAACACAT

GCAAGTCGAACGTGATCCGGAGCTTGCTCCGGTGAAAGTGGCGCACGGGT

GAGTAACGCGTGGATAACCTGCCCTGGTATCTGGGATAACATCTCGAAAG

GGGTGCTAATACCGGATAAGCCTACGGACTCTTCGGAGTCTGCAGGAAAA

GGTGGCCTCTATTTATAAGCTACCGTATCAGGATGGGTCCGCGTACCATT

AGCTAGTTGGTGGGGTAATGGCCTACCAAGGCTACGATGGTTAGCTGGTC

TGAGAGGATGATCAGCCACACTGGAACTGAGACACGGTCCAGACTCCTAC

GGGAGGCAGCAGTGGGGAATTTTGCGCAATGGGGGAAACCCTGACGCAGC

AACGCCGCGTGAGTGATGAAGGCTTTCGGGTCGTAAAGCTCTGTCAGAGG

GGAAGAAATGAGTCTGGCTAATATCCAGATTTCTTGACGGTACCCTCAAA

GGAAGCACCGGCTAACTCCGTGCCAGCAGCCGCGGTAATACGGAGGGTGC

AAGCGTTGTTCGGATTTATTGGGCGTAAAGCGCGTGTAGGCGGTTTTTTA

AGTCTGATGTGAAAGCCCTGGGCTCAACCCAGGAAGTGCATTGGATACTG

GAAGACTTGAATACGGGAGAGGGTAGTGGAATTCCTAGTGTAGGAGTGAA

ATCCGTAGATATTAGGAGGAACACCGGTGGCGAAGGCGGCTACCTGGACC

GATATTGACGCTGAGACGCGAAAGCGTGGGGAGCAAACAGGATTAGATAC

CCTGGTAGTCCACGCCGTAAACGATGAGAACTAGGTGTTGCGGGTATTGA

CCCCTGCAGTGCCGCAGCTAACGCATTAAGTTCTCCGCCTGGGAAGTACG

GTCGCAAGACTAAAACTCAAAGGAATTGACGGGGGCCCGCACAAGCGGTG

GAGCATGTGGTTTAATTCGACGCAACGCGCAGAACCTTACCTGGGCTTGA

CATCTGCGGAATCTTCGTGAAAGCGAGGAGTGCCTTCGGGAGCCGCAAGA

CAGGTGCTGCATGGCTGTCGTCAGCTCGTGTCGTGAGATGTTGGGTTAAG

TCCCGCAACGAGCGCAACCCCTATCCTTAGTTGCCATCATTCAGTTGGGC

ACTCTAAGGAGACTGCCGGTGTCAAACCGGAGGAAGGTGGGGATGACGTC

AAGTCCTCATGGCCCTTATGTCCAGGGCTACACACGTGCTACAATGGCCG

GTACAAAGGGTTGCAATACCGCGAGGTGGAGCCAATCTCAAAAAGCCGGT

CTCAGTTCGGATTGGAGTCTGCAACTCGACTCCATGAAGTTGGAATCGCT

AGTAATCGCGGATCAGCATGCCGCGGTGAATACGTTCCCGGGCCTTGTAC

ACACCGCCCGTCACACCACGGGAGTCGGTTGGTCCCGAAGTGCGTGAGCT

AACCCGCAAGGGAGGCAGCGTCCTAAGGAATGGCCGGTGACTGGGGTGAA

GTCGTAACAAGGTAACCC

>S3_4|CP001124

AAGAGTTTGATCCTGGCTCAGAACGAACGCTGGCGGCGTGCTTAACACAT

GCAAGTCGAACGTGATCCAGAGCTTGCTCTGGTGAAAGTGGCGCACGGGT

GAGTAACGCGTGGATAACCTGCCCTGGTATCTGGGATAACATCTCGAAAG

GGGTGCTAATACCGGATAAGCCTACGGGGTCCTCGGACTCTGCAGGAAAA

GGTGGCCTCTATTTATAAGCTACCGTATCAGGATGGGTCCGCGTACCATT

AGCTAGTTGGTGGGGTAATGGCCCACCAAGGCGACGATGGTTAGCTGGTC

TGAGAGGATGATCAGCCACACTGGAACTGAGACACGGTCCAGACTCCTAC

GGGAGGCAGCAGTGGGGAATTTTGCGCAATGGGGGAAACCCTGACGCAGC

AACGCCGCGTGAGTGATGAAGGCTTTCGGGTCGTAAAGCTCTGTCAGAGG

GGAAGAAATGAGCCTGGCTAATATCCAGGTTTCTTGACGGTACCCTCAAA

GGAAGCACCGGCTAACTCCGTGCCAGCAGCCGCGGTAATACGGAGGGTGC

AAGCGTTGTTCGGATTTATTGGGCGTAAAGCGCGTGTAGGCGGTTTCTTA

AGTCTGATGTGAAAGCCCTGGGCTCAACCCAGGAAGTGCATTGGATACTG

GGAGACTTGAATACGGGAGAGGGTAGTGGAATTCCTAGTGTAGGAGTGAA

ATCCGTAGATATTAGGAGGAACACCGGTGGCGAAGGCGGCTACCTGGACC

GATATTGACGCTGAGACGCGAAAGCGTGGGGAGCAAACAGGATTAGATAC

CCTGGTAGTCCACGCCGTAAACGATGAGAACTAGGTGTTGCGGGTATTGA

CCCCTGCAGTGCCGCAGCTAACGCATTAAGTTCTCCGCCTGGGAAGTACG

GTCGCAAGACTAAAACTCAAAGGAATTGACGGGGGCCCGCACAAGCGGTG

GAGCATGTGGTTTAATTCGACGCAACGCGCAGAACCTTACCTGGGCTTGA

CATCTGCGGAACCCCTGTGAAAGCAGGGGGTGCCTTCGGGAGCCGCAAGA

CAGGTGCTGCATGGCTGTCGTCAGCTCGTGTCGTGAGATGTTGGGTTAAG

TCCCGCAACGAGCGCAACCCCTATCCTTAGTTGCCACCATTTAGTTGGGC

ACTCTAAGGAGACTGCCGGTGTCAAACCGGAGGAAGGTGGGGATGACGTC

AAGTCCTCATGGCCCTTATGTCCAGGGCTACACACGTGCTACAATGGCCG

GTACAAAGGGTTGCAATACCGCGAGGTGGAGCCAATCTCAAAAAGCCGGT

CTCAGTTCGGATTGGAGTCTGCAACTCGACTCCATGAAGTTGGAATCGCT

AGTAATCGCGGATCAGCACGCCGCGGTGAATACGTTCCCGGGCCTTGTAC

ACACCGCCCGTCACACCACGGGAGTCGGTTGGTCCCGAAGTGCGTGAGCT

AACCCGCAAGGGAGGCAGCGTCCTAAGGAATGGCCGGTGACTGGGGTGAA

GTCGTAACAAGGTAACCCCC

>S3_5951|DQ133931

AGAGTTTGATCCTGGCTCAGAACGAACGCTGGCGGCGTGCTTAACACATG

CAAGTCGAACGTGATCCGGAGCTTGCTCCGGTGAAAGTGGCGCACGGGTG

AGTAACGCGTGGATAACCTGCCCTGGTATCTGGGATAACATCTCGAAAGG

GGTGCTAATACCGGATAAGCTCACGACCTCTTCGGAGGATGCGAGAAAAG

GTGGCCTCTATTTATAAGCTACCGTATCATGGGGTCCGCGTACCATTAGC

TAGTTGGTGGGGTAATGGCCTACCAAGGCGACGATGGTTAGCTGGTCTGA

GAGGATGATCAGCCACACTGGAACTGAGACACGGTCCAGACTCCTACGGG

AGGCAGCAGTGGGGAATTTTGCGCAATGGGGGAAACCCTGACGCAGCAAC

GCCGCGTGAGTGATGAAGGCTTTCGGGTCGTAAAGCTCTGTCAGAGGGGA

AGAAATGAGTCTGGCTTAATATCGCGACTTCTTGACGGTACCCTCAAAGG

AAGCACCGGCTAACTCCGTGCCAGCAGCCGCGGTAATACGGAGGGTGCAA

GCGTTGTTCGGATTTATTGGGCGTAAAGCGCGTGTAGGCGGTTTTTTAAG

TCTGATGTGAAAGCCCTGGGCTCAACCCAGGAAGTGCATTGGATACTGGA

AGACTTGAATACGGGAGAGGGTAGTGGAATTCCTAGTGTAGGAGTGAAAT

CCGTAGATATTAGGAGGAACACCGGTGGCGAAGGCGGCTACCTGGACCGA

TATTGACGCTGAGACGCGAAAGCGTGGGGAGCAAACAGGATTAGATACCC

TGGTAGTCCACGCCGTAAACGATGAGAACTAGGTGTTGCGGGTATTGACC

CCTGCAGTGCCGCAGCTAACGCATTAAGTTCTCCGCCTGGGAAGTACGGT

CGCAAGACTAAAACTCAAAGGAATTGACGGGGGCCCGCACAAGCGGTGGA

GCATGTGGTTTAATTCGACGCAACGCGCAGAACCTTACCTGGGCTTGACA

TCTGTGGAACCTCTGTGAAAGCAGGGGGTGCCTTCGGGAGGGAGCCGCAA

GACAGGTGCTGCATGGCTGTCGTCAGCTCGTGTCGTGAGATGTTGGGTTA

AGTCCCGCAACGAGCGCAACCCCTATCCTTAGTTGCCATCATTCAGTTGG

GCACTCTAAGGAGACTGCCGGTGTCAAACCGGAGGAAGGTGGGGATGACG

TCAAGTCCTCATGGCCCTTATGTCCAGGGCTACACACGTGCTACAATGGC

CGGTACAAAGGGTTGCAATACCGCGAGGTGGAGCCAATCTCAAAAAGCCG

GTCTCAGTTCGGATTGGAGTCTGCAACTCGACTCCATGAAGTTGGAATCG

CTAGTAATCGCGGATCAGCATGCACGCGGTGAATACGTTCCCGGGCCTTG

TACACACCGCCCGTCACACCATGGGAGTTTGTTGCACCAGAAGTAGGTAG

TCTAACCGCAAGGAGGACGCTTACCACGGTGTGGCAGATGACTGGGGTGA

AGTCGTAACAAGGTAACC

>S3_1044|FJ902092

TGATCCAGAGCTTGCTCTGGTGAAAGTGGCGCACGGGTGAGTAACGCGTG

GATAACCTGCCCTGGTATCTGGGATAACATCTCGAAAGGGGTGCTAATAC

CGGATAAGCCTACGGGATCTTCGGATTCTGCAGGAAAAGGAGGCCTCTAT

TTATAAGCTTCCGTATCATGATGGGTCCGCGTACCATTAGCTAGTTGGTG

GGGTAATGGCCTACCAAGGCGACGATGGTTAGCTGGTCTGAGAGGATGAT

CAGCCACACTGGAACTGAGACACGGTCCAGACTCCTACGGGAGGCAGCAG

TGGGGAATTTTGCGCAATGGGGGAAACCCTGACGCAGCAACGCCGCGTGA

GTGATGAAGGCTTTCGGGTCGTAAAGCTCTGTCAGAGGGGAAGAAATGAA

ACGTGCTAATATCACGTTTTCTTGACGGTACCCTCAAAGGAAGCACCGGC

TAACTCCGTGCCAGCAGCCGCGGTAATACGGAGGGTGCAAGCGTTGTTCG

GATTTATTGGGCGTAAAGCGCGTGTAGGCGGTTTCTTAAGTCTGATGTGA

AAAGCCCTGGGCTCAACCCAGGAAGTGCATTGGATACTGGGAGACTTGAA

TACGGGAGAGGGTAGTGGAATTCCTAGTGTAGGAGTGAAATCCGTAGATA

TTAGGAGGAACACCGGTGGCGAAGGCGGCTACCTGGACCGATATTGACGC

TGAGACGCGAAAGCGTGGGGAGCAAACAGGATTAGATACCCTGGTAGTCC

ACGCCGTAAACGATGAGAACTAGGTGTTGCGGGTATTGACCCCTGCAGTG

CCGCAGCTAACGCATTAAGTTCTCCGCCTGGGAAGTACGGTCGCAAGACT

AAAACTCAAAGGAATTGACGGGGGCCCGCACAAGCGGTGGAGCATGTGGT

TTAATTCGACGCAACGCGCAGAACCTTACCTGGGCTTGACATCTGCGGAA

TCTTCGTGAAAGCGGGGGGTGCCTTCGGGGGAGCCGTAAGACAGGTGCTG

CATGGCTGTCGTCAGCTCGTGTCGTGAGATGTTGGGTTAAGTCCCGCAAC

GAGCGCAACCCTTGTCTTTAATTGCCATCATTAAGTTGGGCACTTTGGAG

AGACTGCCGGTGTTAAACCGGAGGAAGGTGGGGATGACGTCAAGTCCTCA

TGGCCTTTATGCCCAGGGCTACACACGTGCTACAATGGGTGGTACAAAGA

GCAGCGAACTCGTGAGAGCAAGCGAATCTCAAAAAGCCATCCTCAGTTCG

GATTGAAGTCTGCAACTCGACTTCATGAAGTCGGAATCGCTAGTAATCGC

GGATCAGCATGCCGCGGTGAATACGTTCCCGGGCCTTGTC

>S3_8636|CP002431

AGAGTTTGATCCTGGCTCAGAACGAACGCTGGCGGCGTGCTTAACACATG

CAAGTCGAACGTGATCCAGAGCTTGCTCTGGTGAAAGTGGCGCACGGGTG

ATAACGCGTGGATAACCTGCCCTGGTATCTGGGATAACATCTCGAAAGGG

GTGCTAATACCGGATAAGCCTACGGGATCTTCGGATTCTGCAGGAAAAGG

AGGCCTCTATTTATAAGCTACCGTATCAGGATGGGTCCGCGTACCATTAG

CTAGTTGGTGGGGTAATGGCCTACCAAGGCGACGATGGTTAGCTGGTCTG

AGAGGATGATCAGCCACACTGGAACTGAGACACGGTCCAGACTCCTACGG

GAGGCAGCAGTGGGGAATTTTGCGCAATGGGGGAAACCCTGACGCAGCAA

CGCCGCGTGAGTGATGAAGGCTTTCGGGTCGTAAAGCTCTGTCAGAGGGG

AAGAAAATGAGCCTGGCTAATACCCGTTTTTCTTGACGGTACCCTCAAAG

GAAGCACCGGCTAACTCCGTGCCAGCAGCCGCGGTAATACGGAGGGTGCA

AGCGTTGTTCGGATTTATTGGGCGTAAAGCGCGTGTAGGCGGTTTTTTAA

GTCTGATGTGAAAGCCCTGGGCTCAACCCAGGAAGTGCATTGGATACTGG

AAGACTTGAATACGGGAGAGGGTAGTGGAATTCCTAGTGTAGGAGTGAAA

TCCGTAGATATTAGGAGGAACACCGGTGGCGAAGGCGGCTACCTGGACCG

ATATTGACGCTGAGACGCGAAAGCGTGGGGAGCAAACAGGATTAGATACC

CTGGTAGTCCACGCCGTAAACGATGAGAACTAGGTGTTGGGTCTCCCCTG

CAGTGCCGCAGCTAACGCATTAAGTTCTCCGCCTGGGAAGTACGGTCGCA

AGACTAAAACTCAAAGGAATTGACGGGGGCCCGCACAAGCGGTGGAGCAT

GTGGTTTAATTCGACGCAACGCGCAGAACCTTACCTGGGCTTGACATCTG

CGGAACCCCTGTGAAAGCGAGGAGTGCCTTCGGGGGAGCCGCAAGACAGG

TGCTGCATGGCTGTCGTCAGCTCGTGTCGTGAGATGTTGGGTTAAGTCCC

GCAACGAGCGCAACCCCTATCCTTAGTTGCCATCATTCAGTTGGGCACTC

TAAGGAGACTGTGCCCGGGTCAACCGGGAGGAAGGTGGGGACGACGTCAA

GTCATCATGGCCCTTACGCCTAGGGCTACACACGTACTACAATGGTGCAC

ACAAAGGGCAGCGAGACCGCGAGGTGGAGCCAATCCCAAAAAATGCATCC

CAGTCCGGATTGCAGTCTGCAACTCGACTGCATGAAGTTGGAATCGCTAG

TAATTCCGGATCAGCATGCCGGGGTGAATACGTTCCCGGGCCTTGTACAC

ACCGCCCGTCACACCACGAAAGCTGGTTCTACCCGACAACGGCAGACTAA

CCCTCGGGAGGTAGTCGTCTACGGTAGGGCTGGTAATTGGGGTGAAGTCG

TAACAAGGTAACC

>S6_4|CP001124

AACTGAAGAGTTTGATCCTGGCTCAGAACGAACGCTGGCGGCGTGCTTAA

CACATGCAAGTCGAACGTGATCCAGAGCTTGCTCTGGTGAAAGTGGCGCA

CGGGTGAGTAACGCGTGGATAACCTGCCCTGGTATCTGGGATAACATCTC

GAAAGGGGTGCTAATACCGGATAAGCCTACGGGGTCCTCGGACTCTGCAG

GAAAAGGTGGCCTCTATTTATAAGCTACCGTATCAGGATGGGTCCGCGTA

CCATTAGCTAGTTGGTGGGGTAATGGCCCACCAAGGCGACGATGGTTAGC

TGGTCTGAGAGGATGATCAGCCACACTGGAACTGAGACACGGTCCAGACT

CCTACGGGAGGCAGCAGTGGGGAATTTTGCGCAATGGGGGAAACCCTGAC

GCAGCAACGCCGCGTGAGTGATGAAGGCTTTCGGGTCGTAAAGCTCTGTC

AGAGGGGAAGAAATGAGCCTGGCTAATATCCAGGTTTCTTGACGGTACCC

TCAAAGGAAGCACCGGCTAACTCCGTGCCAGCAGCCGCGGTAATACGGAG

GGTGCAAGCGTTGTTCGGATTTATTGGGCGTAAAGCGCGTGTAGGCGGTT

TCTTAAGTCTGATGTGAAAGCCCTGGGCTCAACCCAGGAAGTGCATTGGA

TACTGGGAGACTTGAATACGGGAGAGGGTAGTGGAATTCCTAGTGTAGGA

GTGAAATCCGTAGATATTAGGAGGAACACCGGTGGCGAAGGCGGCTACCT

GGACCGATATTGACGCTGAGACGCGAAAGCGTGGGGAGCAAACAGGATTA

GATACCCTGGTAGTCCACGCCGTAAACGATGAGAACTAGGTGTTGCGGGT

ATTGACCCCTGCAGTGCCGCAGCTAACGCATTAAGTTCTCCGCCTGGGAA

GTACGGTCGCAAGACTAAAACTCAAAGGAATTGACGGGGGCCCGCACAAG

CGGTGGAGCATGTGGTTTAATTCGACGCAACGCGCAGAACCTTACCTGGG

CTTGACATCTGCGGAACCCCTGTGAAAGCAGGGGGTGCCTTCGGGAGCCG

CAAGACAGGTGCTGCATGGCTGTCGTCAGCTCGTGTCGTGAGATGTTGGG

TTAAGTCCCGCAACGAGCGCAACCCCTATCCTTAGTTGCCACCATTTAGT

TGGGCACTCTAAGGAGACTGCCGGTGTCAAACCGGAGGAAGGTGGGGATG

ACGTCAAGTCCTCATGGCCCTTATGTCCAGGGCTACACACGTGCTACAAT

GGCCGGTACAAAGGGTTGCAATACCGCGAGGTGGAGCCAATCTCAAAAAG

CCGGTCTCAGTTCGGATTGGAGTCTGCAACTCGACTCCATGAAGTTGGAA

TCGCTAGTAATCGCGGATCAGCACGCCGCGGTGAATACGTTCCCGGGCCT

TGTACACACCGCCCGTCACACCACGGGAGTCGGTTGGTCCCGAAGTGCGT

GAGCTAACCCGCAAGGGAGGCAGCGTCCTAAGGAATGGCCGGTGACTGGG

GTGAAGTCGTAACAAGGTAACCC

>S6_8153|JF344521

AGAGTTTGATCCTGGCTCAGAACGAACGCTGGCGGCGTGCTTAACACATG

CAAGTCGAACGTGATCCGGAGCTTGCTCCGGTGAAAGTGGCGCACGGGTG

AGGAGTAACGCGTGGATAACCTGCCCTGGTATCTGGGATAACATCTCGAA

AGGGGTGCTAATACCGGATAAGCCTACGGATTCCTCGGAGTCTGCAGGAA

AAGGTGGCCTCTATTTATAAGCTACCGTATCATGATGGGTCCGCGTACCA

TTAGCTAGTTGGTGGGGTAATGGCCTACCAAGGCGACGATGGTTAGCTGG

TCTGAGAGGATGATCAGCCACACTGGAACTGAGACACGGTCCAGACTCCT

ACGGGAGGCAGCAGTGGGGAATTTTGCGCAATGGGGGAAACCCTGACGCA

GCAACGCCGCGTGAGTGATGAAGGCTTTCGGGTCGTAAAGCTCTGTCAGA

GGGGAAGAAAAATGACGGTACCCTCAAAGGAAGCACCGGCTAACTCCGTG

CCAGCAGCCGCGGTAATACGGAGGGTGCAAGCGTTGTTCGGATTTATTGG

GCGTAAAGCGCGTGTAGGCGGTTTTTTAAGTCTGATGTGAAAGCCCTGGG

CTCAACCCAGGAAGTGCATTGGATACTGGAAGACTTGAATACGGGAGAGG

GTAGTGGAATTCCTAGTGTAGGAGTGAAATCCGTAGATATTAGGAGGAAC

ACCGGTGGCGAAGGCGGCTACCTGGACCGATATTGACGCTGAGACGCGAA

AGCGTGGGGAGCAAACAGGATTAGATACCCTGGTAGTCCACGCCGTAAAC

GATGAGAACTAGGTGTTGCGGGTATTGACCCCTGCAGTGCCGCAGCTAAC

GCATTAAGTTCTCCGCCTGGGAAGTACGGTCGCAAGACTAAAACTCAAAG

GAATTGACGGGGGCCCGCACAAGCGGTGGAGCATGTGGTTTAATTCGACG

CAACGCGCAGAACCTTACCTGGGCTTGACATCTGTGGAACCTCTGTGAAA

GCAGGGGGTGCCTTCGGGAGCCACAAGACAGGTGCTGCATGGCTGTCGTC

AGCTCGTGTCGTGAGATGTTGGGTTAAGTCCCGCAACGAGCGCAACCCCT

ATCCTTAGTTGCCAGCACGTAATGGTGGGAACTCTAAGGAGACTGCCGGT

GTCAAACCGGAGGAAGGTGGGGATGACGTCAAGTCCTCATGGCCTTTATG

TCTAGGGCTACACACGTACTACAATGGCCGGTACAAAGGGCAGCCACTCA

GCGATGAGGCGCTAATCCCATAAAGCCGGTCTCAGTCCGGATCGCAGTCT

GCAACTCGACTGCGTGAAGTTGGAATCGCTAGTAATCGTAGATCAGCATG

CTACGGTGAATACGTTCCCGGGCCTTGTACACACCGCCCGTCACACCACG

AGAGTTGGTTGTTCCAGAAGCCGGTGAGGTAAC

>S6_3844|FJ810552

AGAGTTTGATCCTGGCTCAGAACGAACGCTGGCGGCGTGCTTAACACATG

CAAGTCGAACGTGATCCGGAGCTTGCTCCGGTGAAAGTGGCGCACGGGTG

AGTAGGGTGCTAATACCGGATAAGCCTACGGGATCCTCGGATTCTGCAGG

AAAAGGTGGCCTCTATTTATAAGCTACCGTATCAGGATGAGTCCGCGTAC

CATTAGCTAGTTGGTGGGGTAATGGCCCACCAAGGCTACGATGGTTAGCT

GGTCTGAGAGGATGATCAGCCACACTGGAACTGAGACACGGTCCAGGGGA

ATTTTGCGCAATGGGGGAAACCCTGACGCAGCAACGCCGCGTGAGTGATG

AAGGCTTTCGGGTCGTAAAGCTCTGTCAGAGGGGAAGAAATGAAACGTGC

TAATATCACGTTTTCTTGACGGTACCCTCAAAGGAAGCACCGGCTAACTC

CGTGCCAGCAGCCGCGGTAATACGGAGGGTGCAAGCGTTGTTCGGATTTA

TTGGGCGTAAAGCGCGTGTAGGCGGTTTCTTAAGTCTGATGTGAAAGCCC

TGGGCTCAACCCAGGAAGTGCATTGGATACTGGGAGACTTGAATACGGGA

GAGGGTAGTGGAATTCCTAGTGTAGGAGTGAAATCCGTAGATATTAGGAG

GAACACCGGTGGCGAAGGCGGCTACCTGGACCGATATTGACGCTGAGACG

CGAAAGCGTGGGGAGCAAACAGGATTAGATACCCTGGTAGTCCACGCCGT

AAACGATGAGAACTAGGTGTTGCGGGTATTGACCCCTGCAGTGCCGCAGC

TAACGCATTAAGTTCTCCGCCTGGGAAGTACGGTCGCAAGACTAAAACTC

AAAGGAATTGACGGGGGCCCGCACAAGCGGTGGAGCATGTGGTTTAATTC

GACGCAACGCGCAGAACCTTACCTGGGCTTGACATCTGTGGAACCTCTGT

GAAAGCAGGGGGTGCCTTCGGGAGCCACAAGACAGGTGCTGCATGGCTGT

CGTCAGCTCGTGTCGTGAGATGTTGGGTTAAGTCCCGCAACGAGCGCAAC

CCCTATCCTTAGTTGCCACCATTTAGTTGGGCACTCTAAGGAGACTGCCA

CTGCCCCGGTTAACGGGGAGGAAGGTGGGGACGACGTCAAATCATCATGG

CCCTTACGGCCAGGGCTACACACGTGTTACAATGGGCGCCACAATGGGCA

GCTACTGGGCAACCAGGAGCCAATCTCAAAAAGCCGGTCTCAGTTCGGAT

TGGAGTCTGCAACTCGACTCCATGAAGTTGGAATCGCTAGTAATCGCGGA

TCAGCACGCCGCGGTGAATACGTTCCCGGGCCTTGTACACACCGCCCGTC

ACACCATGGAAGCTGGTGACGCCCGAAATCGGTATCCTAACCGCAAGGAG

GGAGCCGCCTAAGGCAGTGCTGGTGACTGGGGTGAAGTCGTAACAAGGTA

ACC

>S6_476|HQ183850

AGAGTTTGATCCTGGCTCAGAACAAACGCTGGCGGCGTGCCTAACACATG

CAAGTCGTACGTGAAAATCCGCTTCGGTGGATGAGTAAAGTGGCGCACGG

GTGAGTAACGCGTGGAACCTGCCCTGGTATCTGGGATAACATCTCGAAAG

GGGTGCTAATACCGGATAAGCCTACGGATTCCTCGGAGTCTGCAGGAAAA

GGTGGCCTCTATTTATAAGCTACCGTATCATGATGGGTCCGCGTACCATT

AGCTAGTTGGTGGGGTAATGGCCTACCAAGGCGACGATGGTTAGCTGGTC

TGAGAGGATGATCAGCCACACTGGAACTGAGACACGGTCCAGACTCCTAC

GGGAGGCAGCAGTGGGGAATTTTGCGCAATGGGGGAAACCCTGACGCAGC

AACGCCGCGTGAGTGATGAAGGCTTTCGGGTCGTAAAGCTCTGTCAGAGG

GGAAGAAATGGGTCGTGCTAATATCGCGACTTCTTGACGGTACCCTCAAA

GGAAGCACCGGCTAACTCCGTGCCAGCAGCCGCGGTAATACGGAGGGTGC

AAGCGTTGTTCGGATTTATTGGGCGTAAAGCGCGTGTAGGCGGTTTCTTA

AGTCTGATGTGAAAGCCCTGGGCTCAACCCAGGAAGTGCATTGGATACTG

GGAGACTTGAATACGGGAGAGGGTAGTGGAATTCCTAGTGTAGGAGTGAA

ATCCGTAGATATTAGGAGGAACACCGGTGGCGAAGGCGGCTACCTGGACC

GATATTGACGCTGAGACGCGAAAGCGTGGGGAGCAAACAGGATTAGATAC

CCTGGTAGTCCACGCCGTAAACGATGAGAACTAGGTGTTGCGGGTATTGA

CCCCTGCAGTGCCGCAGCTAACGCATTAAGTTCTCCGCCTGGGAAGTACG

GTCGCAAGACTAAAACTCAAAGGAATTGACGGGGGCCCGCACAAGCGGTG

GAGCATGTGGTTTAATTCGACGCAACGCGCAGAACCTTACCTGGGCTTGA

CATCTGCGGAATCTTCGTGAAAGCGAGGGGTGCCTTTCGGGGAGCCGCAA

GACAGGTGCTGCATGGCTGTCGTCAGCTCGTGTCGTGAGATGTTGGGTTA

AGTCCCGCAACGAGCGCAACCCCTATCCTTAGTTGCCATCATTCAGTTGG

GCACTCTAAGGAGACTGCCGGTGTCAAACCGGAGGAAGGTGGGGATGACG

TCAAGTCCTCATGGCCCTTATGTCCAGGGCTACACACGTGCTACAATGGG

TGGTACAAAGAGCAGCAAGCTCGCGAGAGTAAGCGAATCTCAAAAAGCCA

TCCTCAGTTCGGATTGAAGTCTGCAACTCGACTTCATGAAGTTGGAATCG

CTAGTAATCGCGGATCAGCATGCCGCGGTGAATACGTTCCCGGGCCTTGT

ACACACCGCCCGTCACACCCGGAAAATTGATTGCACCAGAAGTCGTTGAG

CTAACCGCTATTTATAGTGGAGGCAGGCGCCTACGGTGTGGTTAGTAATC

GGGGTGAAGTCGTAACAAGGTAACC

>S6_14238|GQ500708

AGAGTTTGATCCTGGCTCAGAACGAACGCTGGCGGCGTGCTTAACACATG

CAAGTCGAACGTGATTCAGAGCTTGCTCTGATGAAAGTGGCGCACGGGTG

AGGTGAGTAACGCGTGGATAACCTGCCCTGGTATCTGGGATAACATCTCG

AAAGGGGTGCTAATACCGGATAAGCCTCCGGGATCTTCGGATTCTGCAGG

AAAAGAAGGCCTCTATTTATAAGCTTTCGTATCAGGATGGGTCCGCGTAC

CATTAGCTAGTTGGTGGGGTAATGGCCTACCAAGGCGACGATGGTTAGCT

GGTCTGAGAGGATGATCAGCCACACTGGAACTGAGACACGGTCCAGACTC

CTACGGGAGGCAGCAGTGGGGAATTTTGCGCAATGGGGGAAACCCTGACG

CAGCAACGCCGCGTGAGTGATGAAGGCTTTCGGGTCGTAAAGCTCTGTCA

GAGGGGAAGAAATGGAAACGGTTAATACCCGTTTTTCTTGACGGTACCCT

CAAAGGAAGCACCGGCTAACTCCGTGCCAGCAGCCGCGGTAATACGGAGG

GTGCAAGCGTTGTTCGGATTTATTGGGCGTAAAGCGCGTGTAGGCGGTTT

CTTAAGTCTGATGTGAAAGCCCTGGGCTCAACCCAGGAAGTGCATTGGAT

ACTGGGAGACTTGAATACGGGAGAGGGTAGTGGAATTCCTAGTGTAGGAG

TGAAATCCGTAGATATTAGGAGGAACACCGGTGGCGAAGGCGGCTACCTG

GACCGATATTGACGCTGAGACGCGAAAGCGTGGGGAGCAAACAGGATTAG

ATACCCTGGTAGTCCACGCCGTAAACGATGAGAACTAGGTGTTGCGGGTA

TTGACCCCTGCAGTGCCGCAGCTAACGCATTAAGTTCTCCGCCTGGGAAG

TACGGTCGCAAGACTAAAACTCAAAGGAATTGACGGGGGCCCGCACAAGC

GGTGGAGCATGTGGTTTAATTCGACGCAACGCGCAGAACCTTACCTGGGC

TTGACATCTGCGGAATCTTCGTGAAAGCGAGGAGTGCCTTCGGGGAGCCG

CCGCAAGACAGGTGCTGCATGGCTGTCGTCAGCTCGTGTCGTGAGATGTT

GGGTTAAGTCCCGCAACGAGCGCAACCCCTATCCTTAGTTGCCATCATTC

AGTTGGGCACTCTAAGGAGACTGCCGGTGATAAACCGGAGGAAGGTGGGG

ATGACGTCAAATCATCATGCCCCTTATGTTCTGGGCTACACACGTGCTAC

AATGGCCAGTACAGACGGAAGCGAAGCCGCGAGGTGGAGCAAATCCGAGA

AAGCTGGTCTCAGTTCGGATTGCAGGCTGCAACTCGCCTGCATGAAGTCG

GAATCGCTAGTAATCGCAGGTCAGCATACTGCGGTGAATACGTTCCCGGG

CCTTGTACACACCGCCCGTCACACCACGAAAGTCTGCAACACCCGAAGCC

GGTGAGGTAACCCGTAAGGGAGCTAGCCGTCGAAGGTGGGGCCGATAATT

GGGGTGAAGTCGTAACAAGGTAACCC

>S6_15078|GQ500708

AGAGTTTGATCCTGGCTCAGAACGAACGCTGGCGGCGTGCTTAACACATG

CAAGTCGAACGTGATCCGGAGCTTGCTCCGGTGAAAGTGGCGCACGGGTG

AGGTGAGTAACGCGTGGATAATCTGCCCTGGTATCTGGGATAACATCTCG

AAAGGGGTGCTAATACCGGATAAGCCCACGGGCTCCTCGGAGTCTGCGGG

AAAAGGTGGCCTCTATTTATAAGCTACCGTATCATGATGGGTCCGCGTAC

CATTAGCTAGTTGGTGGGGTAATGGCCTACCAAGGCGACGATGGTTAGCT

GGTCTGAGAGGATGATCAGCCACACTGGAACTGAGACACGGTCCAGACTC

CTACGGGAGGCAGCAGTGGGGAATTTTGCGCAATGGGGGAAACCCTGACG

CAGCAACGCCGCGTGAGTGATGAAGGCTTTCGGGTCGTAAAGCTCTGTCG

GAGGGGAAGAAATGGAAACGGTTAATACCCGTTTTTCTTGACGGTACCCT

CAAAGGAAGCACCGGCTAACTCCGTGCCAGCAGCCGCGGTAATACGGAGG

GTGCAAGCGTTGTTCGGATTTATTGGGCGTAAAGCGCGTGTAGGCGGTTT

TTTAAGTCTGATGTGAAAGCCCTGGGCTCAACCCAGGAAGTGCATTGGAT

ACTGGAAGACTTGAATACGGGAGAGGGTAGTGGAATTCCTAGTGTAGGAG

TGAAATCCGTAGATATTAGGAGGAACACCGGTGGCGAAGGCGGCTTACTG

GACTGTAACTGACACTGAGGCACGAAAGCGTGGGGAGCGAACAGGATTAG

ATACCCTGGTAGTCCACGCCGTAAACGATGAATACTAGGTGTCGGGGGTC

AAACCTCGGTGCCGCAGCTAACGCAATAAGTATTCCGACCGCCTGGGAAG

TACGGTCGCAAGACTAAAACTCAAAGGAATTGACGGGGGCCCGCACAAGC

GGTGGAGCATGTGGTTTAATTCGACGCAACGCGCAGAACCTTACCTGGGC

TTGACATCTGCGGAATCTTCGTGAAAGCGAGGAGTGCCTTCGGGAGCCGC

AAGACAGACAGGTGCTGCATGGCTGTCGTCAGCTCGTGTCGTGAGATGTT

GGGTTAAGTCCCGCAACGAGCGCAACCCCTATCCTTAGTTGCCATCATTC

AGTTGGGCACTCTAAGGAGACTGCCGGTGTCAAACCGGAGGAAGGTGGGG

ATGACGTCAAGTCCTCATGGCCCTTATGTCCAGGGCTACACACGTGCTAC

AATGGCCGGTACAAAGGGTTGCAATACCGCGAGGTGGAGCCAATCTCAAA

AAGCCGGTCTCAGTTCGGATTGGAGTCTGCAACTCGCCTGCATGAAGTCG

GAATCGCTAGTAATCGCAGGTCAGCATACTGCGGTGAATACGTTCCCGGG

CCTTGTACACACCGCCCGTCACACCACGAAAGTCTGCAACACCCGAAGCC

GGTGAGGTAACCCGTAAGGGAGCTAGCCGTCGAAGGTGGGGCCGATAATT

GGGGTGAAGTCGTAACAAGGTAACCA

>S6_15346|HM748811

AAGAGTTTGATCCTGGCTCAGAACGAACGCTGGCGGCGTGCTTAACACAT

GCAAGTCGAACGTGATTGAGAGCTTGCTCTCATGAAAGTGGCGCACGGGT

GATAACACGTGGATAATCTGCCTGGCGATTCGGGATAACACTTCGAAAGG

GGTGCTAATACCGGATAAGCCCACGGTCTCTTTGGAGAGTGCGGGAAAAG

GGGGCCTCTATTTATAAGCTACCGTATCAGGATGGGTCCGCGTACCATTA

GCTAGTTGGTGGGGTAATGGCCTACCAAGGCGACGATGGTTAGCTGGTCT

GAGAGGATGATCAGCCACACTGGAACTGAGACACGGTCCAGACTCCTACG

GGAGGCAGCAGTGGGGAATTTTGCGCAATGGGGGAAACCCTGACGCAGCA

ACGCCGCGTGAGTGATGAAGGCTTTCGGGTCGTAAAGCTCTGTCAGAGGG

GAAAGAAATGAGCCTGGCTAATATCGCGACTTCTTGACGGTACCCTCAAA

GGAAGCACCGGCTAACTCCGTGCCAGCAGCCGCGGTAATACGGAGGGTGC

AAGCGTTGTTCGGATTTATTGGGCGTAAAGCGCGTGTAGGCGGTTTCTTA

AGTCTGATGTGAAAGCCCTGGGCTCAACCCAGGAAGTGCATTGGATACTG

GGAGACTTGAATACGGGAGAGGGTAGTGGAATTCCTAGTGTAGGAGTGAA

ATCCGTAGATATTAGGAGGAACACCGGTGGCGAAGGCGGCTACCTGGACC

GATATTGACGCTGAGACGCGAAAGCGTGGGGAGCAAACAGGATTAGATAC

CCTGGTAGTCCACGCCGTAAACGATGAGAACTAGGTGTTGCGGGTATTGA

CCCCCTGCAGTGCCGCAGCTAACGCATTAAGTTCTCCGCCTGGGAAGTAC

GGTCGCAAGACTAAAACTCAAAGGAATTGACGGGGGCCCGCACAAGCGGT

GGAGCATGTGGTTTAATTCGACGCAACGCGCAGAACCTTACCTGGGCTTG

ACATCTGTGGAATCTTCGTGAAAGCGAGGAGTGCCTTCGGGAGCCACAAG

ACAGGTGCTGCATGGCTGTCGTCAGCTCGTGTCGTGAGATGTTGGGTTAA

GTCCCGCAACGAGCGCAACCCCTATCCTTAGTTGCCATCATTCAGTTGGG

CACTCTAAGGAGACTGCCGGTGTCAAACCGGAGGAAGGTGGGGATGACGT

CAAGTCCTCATGGCCCTTATGTCCAGGGCTACACACGTGCTACAATGGCC

GGTACAAAGGGTTGCTACCTCGCGAGAGGATGCTAATCTCAAAAAGCCGG

TCGTAGTTCGGATTGGAGTCTGCAACTCGACTCCATGAAGTTGGAATCGC

TAGTAATCGCGGATCAGCACGCCGCGGTGAATACGTTCCCGGGCCTTGTA

CACACCGCCCGTCACACCACGGGAGTCGGTTGGTCCCGAAGTGCGTGAGC

TAACCCGCAAGGGAGGCAGCGTCCTAAGGAATGGCCGGTGACTGGGGTGA

AGTCGTAACAAGGTAACC

>S9_9|CP001124

AAACTGAAGAGTTTGATCCTGGCTCAGAACGAACGCTGGCGGCGTGCTTA

ACACATGCAAGTCGAACGTGATCCGGAGCTTGCTCCGGTGAAAGTGGCGC

ACGGGTGAGTAACGCGTGGATAATCTGCCCTGGTATCTGGGATAACATCT

CGAAAGGGGTGCTAATACCGGATAAGCCTACGGACTCTTCGGAGTCTGCA

GGAAAAGGTGGCCTCTATTTATAAGCTACCGTATCAGGATGAGTCCGCGT

ACCATTAGCTAGTTGGTGGGGTAATGGCCCACCAAGGCTACGATGGTTAG

CTGGTCTGAGAGGATGATCAGCCACACTGGAACTGAGACACGGTCCAGAC

TCCTACGGGAGGCAGCAGTGGGGAATTTTGCGCAATGGGGGAAACCCTGA

CGCAGCAACGCCGCGTGAGTGATGAAGGCTTTCGGGTCGTAAAGCTCTGT

CAGAGGGGAAGAAATGAGACTGGCTAATATCCAGTTTTCTTGACGGTACC

CTCAAAGGAAGCACCGGCTAACTCCGTGCCAGCAGCCGCGGTAATACGGA

GGGTGCAAGCGTTGTTCGGATTTATTGGGCGTAAAGCGCGTGTAGGCGGT

TTCTTAAGTCTGATGTGAAAGCCCTGGGCTCAACCCAGGAAGTGCATTGG

ATACTGGGAGACTTGAATACGGGAGAGGGTAGTGGAATTCCTAGTGTAGG

AGTGAAATCCGTAGATATTAGGAGGAACACCGGTGGCGAAGGCGGCTACC

TGGACCGATATTGACGCTGAGACGCGAAAGCGTGGGGAGCAAACAGGATT

AGATACCCTGGTAGTCCACGCCGTAAACGATGAGAACTAGGTGTTGCGGG

TATTGACCCCTGCAGTGCCGCAGCTAACGCATTAAGTTCTCCGCCTGGGA

AGTACGGTCGCAAGACTAAAACTCAAAGGAATTGACGGGGGCCCGCACAA

GCGGTGGAGCATGTGGTTTAATTCGACGCAACGCGCAGAACCTTACCTGG

GCTTGACATCTGTGGAACCTCTGTGAAAGCAGGGGGTGCCTTCGGGAGCC

ACAAGACAGGTGCTGCATGGCTGTCGTCAGCTCGTGTCGTGAGATGTTGG

GTTAAGTCCCGCAACGAGCGCAACCCCTATCCTTAGTTGCCACCATTTAG

TTGGGCACTCTAAGGAGACTGCCGGTGTCAAACCGGAGGAAGGTGGGGAT

GACGTCAAGTCCTCATGGCCCTTATGTCCAGGGCTACACACGTGCTACAA

TGGCCGGTACAAAGGGTTGCAATACCGCGAGGTGGAGCCAATCTCAAAAA

GCCGGTCTCAGTTCGGATTGGAGTCTGCAACTCGACTCCATGAAGTTGGA

ATCGCTAGTAATCGCGGATCAGCACGCCGCGGTGAATACGTTCCCGGGCC

TTGTACACACCGCCCGTCACACCACGGGAGTCGGTTGGTCCCGAAGTGCG

TGAGCTAACCCGCAAGGGAGGCAGCGTCCTAAGGAATGGCCGGTGACTGG

GGTGAAGTCGTAACAAGGTAACCATAGGGGAACCT

>S9_18296|FR667779

AGAGTTTGATCCTGGCTCAGAACGAACGCTGGCGGCGTGCCTAACACATG

CAAGTCGAACGGAATTAAGGGGCTTGCTCCTTAATTTAGTGGCGCACGGG

TGAGTAACGCGTAGATAATCTGCCTGATGATCTGGGATAACACTTCGAAA

GGGGTGCTAATACCGGATAAGCCCACAGGATCTTTGGATCCAGCGGGAAA

AGGGGGGGACCTTCGGGCCTTCTGTCATCAGATGAGTCTGCGTACCATTA

GCTAGTTGGTAGGGTAATGGCCTACCAAGGCTACGATGGTTAGCTGGTCT

GAGAGGATGATCAGCCACACTGGAACTGAGACACGGTCCAGACTCCTACG

GGAGGCAGCAGTGGGGAATTTTGCGCAATGGGCGAAAGCCTGACGCAGCA

ACGCCGCGTGAGTGATGAAGGCTTTCGGGTCGTAAAGCTCTGTCAGAGGG

GAAGAAATGAGACTGGCTAATATCCAGAAAAAAATGACGGTACCCTCAAA

GGAAGCACCGGCTAACTCCGTGCCAGCAGCCGCGGTAATACGGAGGGTGC

AAGCGTTGTTCGGATTTATTGGGCGTAAAGCGCGTGTAGGCGGTTTTTTA

AGTCTGATGTGAAAGCCCTGGGCTCAACCCAGGAAGTGCATTGGATACTG

GAAGACTTGAATACGGGAGAGGGTAGTGGAATTCCTAGTGTAGGAGTGAA

ATCCGTAGATATTAGGAGGAACACCCGGTGGCGAAGGCGGCTACCTGGAC

CGATATTGACGCTGAGACGCGAAAGCGTGGGGAGCAAACAGGATTAGATA

CCCTGGTAGTCCACGCCGTAAACGATGAGAACTAGGTGTTGCGGGTATTG

ACCCCTGCAGTGCCGCAGCTAACGCATTAAGTTCTCCGCCTGGGAAGTAC

GGTCGCAAGACTAAAACTCAAAGGAATTGACGGGGGCCCGCACAAGCGGT

GGAGCATGTGGTTTAATTCGACGCAACGCGCAGAACCTTACCTGGGCTTG

ACATCTGCGGAATCTTCGTGAAAGCGAGGAGTGCCTTCGGGAGCCGCAAG

ACAGGTGCTGCATGGCTGTCGTCAGCTCGTGTCGTGAGATGTTGGGTTAA

GTCCCGCAACGAGCGCAACCCCTATCCTTAGTTGCCATCATTCAGTTGGG

CACTCTAAGGAGACTGCCGGTGTCAAACCGGAGGAAGGTGGGGATGACGT

CAAGTCCTCATGGCCCTTATGTCCAGGGCTACACACGTGCTACAATGGCC

GGTACAAAGGGTTGCAATACCGCGAGGTGGAGCCAATCTCAAAAAGCCGG

TCTCAGTTCGGATTGGAGTCTGCAACTCGACTCCATGAAGTTGGAATCGC

TAGTAATCGCGGATCAGCATGCCGCGGTGAATACGTTCCCGGGCCTTGTA

CACACCGCCCGTCACACCACGGGAGTCGATTGGTCCCGAAGTGCGTGAGC

TAACCCGCAAGGGAGGCAGCGTCCTAAGGAATGGTCGGTGACTGGGGTGA

AGTCGTAACAAGGTAACCC

>S9_18109|FJ793176

AAGAGTTTGATCCTGGCTCAGAACGAACGCTGGCGGCGTGCCTAACACAT

GCAAGTCGAACGGGGATGGGGAGCTTGCTTCCTATTCTAGTGGCGCACGG

GTGAGTAACGCGTAGATAATCTGCCTGACGATCTGGGATAACACTTCGAA

AGGGGTGCTAATACCGGATAAGCCCACGGATTCTTTGGAGTTTGCGGGAA

AAGGGGGGGACCTTTTGGCCTTCTGTCGTCAGATGAGTCTGCGTACCATT

AGCTAGTTGGTAGGGTAAAGGCCTACCAAGGCTACGATGGTTAGCTGGTC

TGAGAGGATGATCAGCCACACTGGAACTGAGACACGGTCCAGACTCCTAC

GGGAGGCAGCAGTGGGGAATTTTGCGCAATGGGCGAAAGCCTGACGCAGC

AACGCCGCGTGAGTGATGAAGGCTTTCGGGTCGTAAAGCTCTGTCGAGGG

GAAAGAAGTGTATTGTGGCTAATATCCATGATACTTGACGGTACCCCTAA

AGGAAGCACCGGCTAACTCCGTGCCAGCAGCCGCGGTAATACGGGGGGTG

CAAGCGTTGTTCGGAATTATTGGGCGTAAAGCGCGTGTAGGCGGTTTGTT

AAGTCTGATGTGAAAGCCCTGGGCTCAACCTGGGAACTGCGTTTGTGACT

GGAAGGCTAGAGTACGGGAGAGGGTAGTGGAATTCCTAGTGTAGGAGTGA

AATCCGTAGATATTAGGAGGAACACCGGTGGCGAAGGCGGCTACCTGGAC

CGATATTGACGCTGAGACGCGAAAGCGTGGGGAGCAAACAGGATTAGATA

CCCTGGTAGTCCACGCCGTAAACGATGAGAACTAGGTGTTGCGGGTATTG

ACCCCTGCAGTGCCGCAGCTAACGCATTAAGTTCTCCGCCTGGGAAGTAC

GGTCGCAAGACTAAAACTCAAAGGAATTGACGGGGGCCCGCACAAGCGGT

GGAGCATGTGGTTTAATTCGACGCAACGCGCAGAACCTTACCTGGGCTTG

ACATCTGCGGAACCCCTGTGAAAGCAGGGGGTGCCTTCGGGAGCCGCAAG

ACAGGTGCTGCATGGCTGTCGTCAGCTCGTGTCGTGAGATGTTGGGTTAA

GTCCCGCAACGAGCGCAACCCCTACCCTCAGTTGCCATCATTAAGTTGGG

CACTCTGTGGGGACTGCCGGTGTCAAACCGGAGGAAGGTGGGGATGACGT

CAAGTCCTCATGGCCCTTATGTCCAGGGCTACACACGTGCTACAATGGCC

GGTACAAAGAGTTGCGATACCGCGAGGTGGAGCCAATCTCATAAAGCCGG

TCTCAGTTCGGATTGGAGTCTGCAACTCGACTCCATGAAGTTGGAATCGC

TAGTAATCGCGGATCAGCATGCCGCGGTGAATACGTTCCCGGGCCTTGTA

CACACCGCCCGTCACACCACGGGAGTCGATTGGTCCCGAAGTGCGTGAGC

TAACCCGTAAGGGAGGCAGCGTCCTAAGGAATGGTCGGTGACTGGGGTGA

AGTCGTAACAAGGTAACCA

>S9_20170|JF736631

AGAGTTTGATCCTGGCTCAGAACGAACGCTGGCGGCGTGCTTAACACATG

CAAGTCGAACGTGATCCAGAGCTTGCTCTGGTGAAAGTGGCGCACGGGTG

AGTAACGCGTGGATAACCTGCCCTGGTATCTGGGATAACATCTCGAAAGG

GGTGCTAATACCGGATAAGCCTACGGACTCCTCGGAGTCTGCAGGAAAAG

GGGGGGACCTTCGGGCCTTCTGTCATCAGATGGGTCCGCGTACCATTAGC

TAGTTGGTGGGGTAATGGCCTACCAAGGCGACGATGGTTAGCTGGTCTGA

GAGGATGATCAGCCACACTGGAACTGAGACACGGTCCAGACTCCTACGGG

AGGCAGCAGTGGGGAATTTTGCGCAATGGGGGAAACCCTGACGCAGCAAC

GCCGCGTGAGTGATGAAGGCTTTCGGGTCGTAAAGCTCTGTCAGAGGGGA

AGAAATGAAACGTGCTAATATCACGTTTTCTTGACGGTACCCTCAGGAAG

AAGCACCGGCTAACTCCGTGCCAGCAGCCGCGGTAATACGGAGGGTGCAA

GCGTTGTTCGGATTTATTGGGCGTAAAGCGCGTGTAGGCGGTTTTTTAAG

TCTGATGTGAAAGCCCTGGGCTCAACCCAGGAAGTGCATTGGATACTGGA

AGACTTGAATACGGGAGAGGGTAGTGGAATTCCTAGTGTAGGAGTGAAAT

CCGTAGATATTAGGAGGAACACCGGTGGCGAAGGCGGCTACCTGGACCGA

TATTGACGCTGAGACGCGAAAGCGTGGGGAGCAAACAGGATTAGATACCC

TGGTAGTCCACGCCGTAAACGATGAGAACTAGGTGTTGCGGGTATTGACC

CCTGCAGTGCCGCAGCTAACGCATTAAGTTCTCCGCCTGGGAAGTACGGT

CGCAAGACTAAAACTCAAAGGAATTGACGGGGGCCCGCACAAGCGGTGGA

GCATGTGGTTTAATTCGACGCAACGCGCAGAACCTTACCTGGGCTTGACA

TCTGCGGAACCCCTGTGAAAGCAGGGGGTGCCTTCGGGAGCCGCAAGACA

GGTGCTGCATGGCTGTCGTCAGCTCGTGTCGTGAGATGTTGGGTTAAGTC

CCGCAACGAGCGCAACCCCTATCCTTAGTTGCCACCATTAAGTTGGGCAC

TCTAGCGAGACTGCCTGGGTTAACCAGGAGGAAGGTGGGGATGACGTCAA

ATCATCATGCCCCTTATGTCTAGGGCTACACACGTGCTACAATGGCCGGT

ACAACGAGAAGCAAACCCGTGAGGGGGAGCCAAACTAAAAAAGCCGTCCT

CAGTTCGGATTGGAGTCTGCAACTCGACTCCATGAAGTTGGAATCGCTAG

TAATCGCGGATCAGCACGCCGCGGTGAATACGTTCCCGGGCCTTGTACAC

ACCGCCCGTCACACCACGGGAGTCGGTTGGTCCCGAAGTGCGTGAGCTAA

CCCGCAAGGGAGGCAGCGTCCTAAGGAATGGCCGGTGACTGGGGTGAAGT

CGTAACAAGGTAACCT

>S9_20605|EU236221

AGAGTTTGATCCTGGCTCAGAACGAACGCTGGCGGCGTGCTTAACACATG

CAAGTCGAACGTGATCCGGAGCTTGCTCCGGTGAAAGTGGCGCACGGGTG

AGTAACGCGTGGATAATCTGCCCTGGTATCTGGGATAACATCTCGAAAGG

GGTGCTAATACCGGATAAGCCTACGGGATCCTCGGATTCTGCAGGAAAAG

GTGGCCTCTGTATATGCTCCTGTCGCTAGATGAGTCCGCGTACCATTAGC

TAGTTGGTGGGGTAATGGCCTACCAAGGCGACGATGGTTAGCTGGTCTGA

GAGGATGATCAGCCACACTGGAACTGAGACACGGTCCAGACTCCTACGGG

AGGCAGCAGTGGGGAATTTTGCGCAATGGGGGAAACCCTGACGCAGCAAC

GCCGCGTGGAGGATGAAGGTCTTCGGATCGTAAACTTCTCTGTCGAGGGG

AAGAAATGAGTCTGGCTAATATCCAGATTTCTTGACGGTACCCTCAAAGG

AAGCACCGGCTAACTCCGTGCCAGCAGCCGCGGTAATACGGAGGGTGCAA

GCGTTGTTCGGATTTATTGGGCGTAAAGCGCGTGTAGGCGGTTTTTTAAG

TCTGATGTGAAAGCCCTGGGCTCAACCCAGGAAGTGCATTGGATACTGGA

AGACTTGAATACGGGAGAGGGTAGTGGAATTCCTAGTGTAGGAGTGAAAT

CCGTAGATATTAGGAGGAACACCGGTGGCGAAGGCGGCTACCTGGACCGA

TATTGACGCTGAGACGCGAAAGCGTGGGGAGCAAACAGGATTAGATACCC

TGGTAGTCCACGCCGTAAACGATGAGAACTAGGTGTTGCGGGTATTGACC

CCTGCAGTGCCGCAGCTAACGCATTAAGTTCTCCGCCTGGGAAGTACGGT

CGCAAGACTAAAACTCAAAGGAATTGACGGGGGCCCGCACAAGCGGTGGA

GCATGTGGTTTAATTCGACGCAACGCGCAGAACCTTACCTGGGCTTGACA

TCTACGGAACCTTCGTGAAAGCGAGGGGTGCCTTTCGGGGAACCGTAAGA

CAGGTGCTGCATGGCTGTCGTCAGCTCGTGTCGTGAGATGTTGGGTTAAG

TCCCGCAACGAGCGCAACCCCTATCCTCAGTTGCCATCATTAAGTTGGGC

ACTCTGTGGAGACTGCCGGTGTCAAACCGGAGGAAGGTGGGGATGACGTC

AAGTCCTCATGGCCCTTATGTCCAGGGCTACACACGTGCTACAATGGCCG

GTACAAAGAGTTGCGATACCGCGAGGTGGAGCCAATCTCATAAAGCCGGT

CTCAGTTCGGATTGGAGTCTGCAACTCGACTCCATGAAGTTGGAATCGCT

AGTAATCGCGGATCAGCATGCCGCGGTGAATACGTTCCCGGGCCTTGTAC

ACACCGCCCGT

>S9_936|EU244081

AGAGTTTGATCCTGGCTCAGAACGAACGCTGGCGGCGTGCCTAACACATG

CAAGTCGAACGGAATTAAGGGGCTTGCTCCTTAATTTAGTGGCGCACGGG

TGAGTAACGCGTAGATAATCTGCCTGATGATCTGGGATAACACTTCGAAA

GGGGTGCTAATACCGGATAAGCCCACAGGATCTTTGGATCCAGAGGAAAA

GGTGGCCTCTATTTATAAGCTACCGTATCAGGATGAGTCTGCGTACCATT

AGCTAGTTGGTAGGGTAATGGCCTACCAAGGCTACGATGGTTAGCTGGTC

TGAGAGGATGATCAGCCACACTGGAACTGAGACACGGTCCAGACTCCTAC

GGGAGGCAGCAGTGGGGAATTTTGCGCAATGGGCGAAAGCCTGACGCAGC

AACGCCGCGTGAGTGATGAAGGCTTTCGGGTCGTAAAGCTCTGTCAAGGG

GAAAGAAGTGTATTGCAGCTAATATCTGCGATACTTGACGGTACCCCTAA

AGGAAGCACCGGCTAACTCCGTGCCAGCAGCCGCGGTAATACGGGGGGTG

CAAGCGTTGTTCGGAATTATTGGGCGTAAAGCGCGTGTAGGCGGTTTGTT

AAGTCTGATGTGAAAGCCCTGGGCTCAACCCAGGAAGTGCATTGGATACT

GGCAGACTTGAATACGGGAGAGGGTAGTGGAATTCCTGGTGTAGGAGTGA

AATCCGTAGATATCAGGAGGAACACCGGTGGCGAAGGCGGCTACCTGGAC

CGATATTGACGCTGAGACGCGAAAGCGTGGGTAGCAAACAGGATTAGATA

CCCTGGTAGTCCACGCCGTAAACGATGAGTACTAGGTGTTGCGGGTATTG

ACCCCTCAGTGCCGCAGCTAACGCATTAAGTACTCCGCCTGGGAAGTACG

GTCGCAAGACTAAAACTCAAAGGAATTGACGGGGGCCCGCACAAGCGGTG

GAGCATGTGGTTTAATTCGACGCAACGCGCAGAACCTTACCTGGGCTTGA

CATCTACGGAAGATTCCAGAGATGGGATTGTGCCTTCGGGAGCCGCAAGA

CAGGTGCTGCATGGCTGTCGTCAGCTCGTGTCGTGAGATGTTGGGTTAAG

TCCCGCAACGAGCGCAACCCCTATCCTTAGTTGCCAGCGAGTAATGTCGG

GCACTCTAAGGAGACTGCCGGTGTCAAACCGGAGGAAGGTGGGGATGACG

TCAAGTCCTCATGGCCCTTATGTCCAGGGCTACACACGTGCTACAATGGC

CGGTACAAAGGGTTGCAATACCGCGAGGTGGAGCCAATCTCAAAAAGCCG

GTCTCAGTTCGGATTGTAGGCTGAAACTCGCCCACGTGAAGTTGGAATCG

CTAGTAATCGCGGATCAGCACGCCGCGGTGAATACGTTCCCGGGCCTTGT

ACACACCGCCCGTCACACCACGGGAGTCGGTTGGTCCCGAAGTGCGTGAG

CTAACCCGCAAGGGAGGCAGCGTCC

>S9_18161|AY780557

AGAGTTTGATCCTGGCTCAGAACGAACGCTGGCGGCGTGCCTAACACATG

CAAGTCGAACGGAATTAAGGGGCTTGCTCCTTAATTTAGTGGCGCACGGG

TGAGTAACGCGTAGATAATCTGCCTGATGATCTGGGATAACACTTCGAAA

GGGGTGCTAATACCGGATAAGCCTACGGACTCTTCGGAGTCTGCAGGAAA

AGGTGGCCTCTTCGGGCCTTCTGTCATCAGGATGGGTCCGCGTACCATTA

GCTAGTTGGTGGGGTAATGGCCTACCAAGGCGACGATGGTTAGCTGGTCT

GAGAGGATGATCAGCCACACTGGAACTGAGACACGGTCCAGACTCCTACG

GGAGGCAGCAGTGGGGAATTTTGCGCAATGGGGGAAACCCTGACGCAGCA

ACGCCGCGTGAGTGATGAAGGCTTTCGGGTCGTAAAGCTCTGTCAGAGGG

GAAGAAATGGAAACGGTTAATACCCGTTTTTCTTGACGGTACCCTCAAAG

GAAGCACCGGCTAACTCCGTGCCAGCAGCCGCGGTAATACGGAGGGTGCA

AGCGTTGTTCGGATTTATTGGGCGTAAAGCGCGTGTAGGCGGTTTCTTAA

GTCTGATGTGAAAGCCCTGGGCTCAACCCAGGAAGTGCATTGGATACTGG

GAGACTTGAATACGGGAGAGGGTAGTGGAATTCCTAGTGTAGGAGTGAAA

TCCGTAGATATTAGGAGGAACACCGGTGGCGAAGGCGGCTACCTGGACCG

ATATTGACGCTGAGACGCGAAAGCGTGGGGAGCAAACAGGATTAGATACC

CTGGTAGTCCACGCCGTAAACGATGAGAACTAGGTGTTGGGGGCATAAAA

CCTGCAGTGCCGAAGCTAACGCATTAAGTATCCCGCCTGGGAAGTACGGT

CGCAAGACTAAAACTCAAAGGAATTGACGGGGGCCCGCACAAGCGGTGGA

GCATGTGGTTTAATTCGACGCAACGCGCAGAACCTTACCTGGGCTTGACA

TGCCAGGAAGTTTAGAGATGGAGGGGTGCCTTTCGGGGAGCCGCAAGACA

GGTGCTGCATGGCTGTCGTCAGCTCGTGTCGTGAGATGTTGGGTTAAGTC

CCGCAACGAGCGCAACCCCTATCCTTAGTTGCCATCATTCAGTTGGGCAC

TCTAAGGAGACTGCCGGTGTCAAACCGGAGGAAGGTGGGGATGACGTCAA

GTCCTCATGGCCCTTATGTCCAGGGCTACACACGTGCTACAATGGCCGGT

ACAAAGAGTTGCGATGCCGCGAGGTGGAGCCAATCTCAAAAAGCCGGTCT

CAGTTCGGATTGGAGTCTGCAACTCGACTCCATGAAGTTGGAATCGCTAG

TAATCGCGGATCAGCATGCCGCGGTGAATACGTTCCCGGGCCTTGTACAC

ACCGCCCGTCACACCACGGGAGTCGGTTGGTCCCGAAGTGCGTGAGCTAA

CCCGCAAGGGAGGCAGCGTCCTAAGGAATGGCCGGTGACTGGGGTGAAGT

CGTAACAAGGTAACC

>S10_51|CP00112

TAAGAGTTTGATCCTGGCTCAGAACGAACGCTGGCGGCGTGCTTAACACA

TGCAAGTCGAACGTGATCCGGAGCTTGCTCCGGTGAAAGTGGCGCACGGG

TGAGTAACGCGTGGATAATCTGCCCTGGTATCTGGGATAACATCTCGAAA

GGGGTGCTAATACCGGATAAGCCTACGGACTCTTCGGAGTCTGCAGGAAA

AGGTGGCCTCTATTTATAAGCTACCGTATCAGGATGAGTCCGCGTACCAT

TAGCTAGTTGGTGGGGTAATGGCCCACCAAGGCTACGATGGTTAGCTGGT

CTGAGAGGATGATCAGCCACACTGGAACTGAGACACGGTCCAGACTCCTA

CGGGAGGCAGCAGTGGGGAATTTTGCGCAATGGGGGAAACCCTGACGCAG

CAACGCCGCGTGAGTGATGAAGGCTTTCGGGTCGTAAAGCTCTGTCAGAG

GGGAAGAAATGAGACTGGCTAATATCCAGTTTTCTTGACGGTACCCTCAA

AGGAAGCACCGGCTAACTCCGTGCCAGCAGCCGCGGTAATACGGAGGGTG

CAAGCGTTGTTCGGATTTATTGGGCGTAAAGCGCGTGTAGGCGGTTTCTT

AAGTCTGATGTGAAAGCCCTGGGCTCAACCCAGGAAGTGCATTGGATACT

GGGAGACTTGAATACGGGAGAGGGTAGTGGAATTCCTAGTGTAGGAGTGA

AATCCGTAGATATTAGGAGGAACACCGGTGGCGAAGGCGGCTACCTGGAC

CGATATTGACGCTGAGACGCGAAAGCGTGGGGAGCAAACAGGATTAGATA

CCCTGGTAGTCCACGCCGTAAACGATGAGAACTAGGTGTTGCGGGTATTG

ACCCCTGCAGTGCCGCAGCTAACGCATTAAGTTCTCCGCCTGGGAAGTAC

GGTCGCAAGACTAAAACTCAAAGGAATTGACGGGGGCCCGCACAAGCGGT

GGAGCATGTGGTTTAATTCGACGCAACGCGCAGAACCTTACCTGGGCTTG

ACATCTGTGGAACCTCTGTGAAAGCAGGGGGTGCCTTCGGGAGCCACAAG

ACAGGTGCTGCATGGCTGTCGTCAGCTCGTGTCGTGAGATGTTGGGTTAA

GTCCCGCAACGAGCGCAACCCCTATCCTTAGTTGCCACCATTTAGTTGGG

CACTCTAAGGAGACTGCCGGTGTCAAACCGGAGGAAGGTGGGGATGACGT

CAAGTCCTCATGGCCCTTATGTCCAGGGCTACACACGTGCTACAATGGCC

GGTACAAAGGGTTGCAATACCGCGAGGTGGAGCCAATCTCAAAAAGCCGG

TCTCAGTTCGGATTGGAGTCTGCAACTCGACTCCATGAAGTTGGAATCGC

TAGTAATCGCGGATCAGCACGCCGCGGTGAATACGTTCCCGGGCCTTGTA

CACACCGCCCGTCACACCACGGGAGTCGGTTGGTCCCGAAGTGCGTGAGC

TAACCCGCAAGGGAGGCAGCGTCCTAAGGAATGGCCGGTGACTGGGGTGA

AGTCGTAACAAGGTAACCC

>S10_71|FR667782

AGAGTTTGATCCTGGCTCAGAACGAACGCTGGCGGCGTGCCTAACACATG

CAAGTCGAACGGAATGGAGGGGCTTGCTCCTTCATTTAGTGGCGCACGGG

TGAGTAACGCGTAGATAATCTGCCTGATGATCCGGGATAACACTTCGAAA

GGGGTGCTAATACCGGATAAGCCCACGACGGCTTTGGTCGTTGCGGGAAA

AGGGGGGGACCTTCGGGCCTTCTGTCATCAGATGAGTCTGCGTACCATTA

GCTAGTTGGTAGGGTAATGGCCTACCAAGGCTACGATGGTTAGCTGGTCT

GAGAGGATGATCAGCCACACTGGAACTGAGACACGGTCCAGACTCCTACG

GGAGGCAGCAGTGGGGAATTTTGCGCAATGGGCGAAAGCCTGACGCAGCA

ACGCCGCGTGAGTGATGAAGGCTTTCGGGTCGTAAAGCTCTGTCAAGGGG

AAAGAAGTGTATTGCAGCTAATATCTGCGATACTTGACGGTACCCCTAAA

GGAAGCACCGGCTAACTCCGTGCCAGCAGCCGCGGTAATACGGGGGGTGC

AAGCGTTGTTCGGAATTATTGGGCGTAAAGCGCGTGTAGGCGGTTTGTTA

AGTCTGATGTGAAAGCCCTGGGCTCAACCCAGGAAGTGCATTGGATACTG

GCAGACTTGAATACGGGAGAGGGTAGTGGAATTCCTGGTGTAGGAGTGAA

ATCCGTAGATATCAGGAGGAACACCCGGTGGCGAAGGCGGCTACCTGGAC

CGATATTGACGCTGAGACGCGAAAGCGTGGGTAGCAAACAGGATTAGATA

CCCTGGTAGTCCACGCCGTAAACGATGAGTACTAGGTGTTGCGGGTATTG

ACCCCTGCAGTGCCGCAGCTAACGCATTAAGTACTCCGCCTGGGAAGTAC

GGTCGCAAGACTAAAACTCAAAGGAATTGACGGGGGCCCGCACAAGCGGT

GGAGCATGTGGTTTAATTCGACGCAACGCGCAGAACCTTACCTGGGCTTG

ACATCTACGGAACCTTCGTGAAAGCGAGGGGTGCCTTTCGGGGAGCCGTA

AGACAGGTGCTGCATGGCTGTCGTCAGCTCGTGTCGTGAGATGTTGGGTT

AAGTCCCGCAACGAGCGCAACCCCTACCCTCAGTTGCCATCATTAAGTTG

GGCACTCTGTGGGGACTGCCGGTGTCAAACCGGAGGAAGGTGGGGATGAC

GTCAAGTCCTCATGGCCCTTATGTCCAGGGCTACACACGTGCTACAATGG

CCGGTACAAAGAGTTGCGATACCGCGAGGTGGAGCCAATCTCATAAAGCC

GGTCTCAGTTCGGATTGGAGTCTGCAACTCGACTCCATGAAGTTGGAATC

GCTAGTAATCGCGGATCAGCATGCCGCGGTGAATACGTTCCCGGGCCTTG

TACACACCGCCCGTCACACCACGGGAGTCGATTGGTCCCGAAGTGCGTGA

GCTAACCCGCAAGGGAGGCAGCGTCCTAAGGAATGGTCGGTGACTGGGGT

GAAGTCGTAACAAGGTAACCC

>S10_475|AY013648

AAGAGTTTGATCCTGGCTCAGAACGAACGCTGGCGGCGTGCCTAACACAT

GCAAGTCGAACGGAATTAAGGGGCTTGCTCCTTAATTTAGTGGCGCACGG

GTGAGTAACGCGTAGATAATCTGCCTGATGATCTGGGATAACACTTCGAA

AGGGGTGCTAATACCGGATAAGCCCACAGGATCTTTGGATCCAGCGGGAA

AAGGGGGGGACCTTCGGGCCTTCTGTCATCAGATGAGTCTGCGTACCATT

AGCTAGTTGGTAGGGTAATGGCCTACCAAGGCTACGATGGTTAGCTGGTC

TGAGAGGATGATCAGCCACACTGGAACTGAGACACGGTCCAGACTCCTAC

GGGAGGCAGCAGTGGGGAATTTTGCGCAATGGGCGAAAGCCTGACGCAGC

AACGCCGCGTGAGTGATGAAGGCTTTCGGGTCGTAAAGCTCTGTCGAGGG

GAAAGAAATGTACGAGGGCTAATATCCTTTGTACTTGACGGTACCCCTAA

AGGAAGCACCGGCTAACTCCGTGCCAGCAGCCGCGGTAATACGGGGGTGC

AAGCGTTGTTCGGAATTATTGGGCGTAAAGCGCGTGTAGGCGGTTTGTTA

AGTCTGATGTGAAAGCCCTGGGCTCAACCCAGGAAGTGCATTGGAAACTG

GCAGACTTGAATACGGGAGAGGGTAGTGGAATTCCTGGTGTAGGAGTGAA

ATCCGTAGATATCAGGAGGAACACCGGTGGCGAAGGCGGCTACCTGGACC

GATATTGACGCTGAGACGCGAAAGCGTGGGTAGCAAACAGGATTAGATAC

CCTGGTAGTCCACGCCGTAAACGATGAGTACTAGGTGTTGCGGGTATTGA

CCCCTGCAGTGCCGCAGCTAACGCATTAAGTACTCCGCCTGGGAAGTACG

GTCGCAAGACTAAAACTCAAAGGAATTGACGGGGGCCCGCACAAGCGGTG

GAGCATGTGGTTTAATTCGACGCAACGCGCAGAACCTTACCTGGGCTTGA

CATCTACGGAACCTTCGTGAAAGCGAGGGGTGCCTTTCGGGGAGCCGTAA

GACAGGTGCTGCATGGCTGTCGTCAGCTCGTGTCGTGAGATGTTGGGTTA

AGTCCCGCAACGAGCGCAACCCTTGTCCTTAGTTGCTACATTCAGTTGGG

CACTCTAATGAGACTGCCGGTGACAAACCGGAGGAAGGTGGGGATGACGT

CAAGTCCTCATGGCCCTTATGGGTAGGGCTTCACACGTAATACAATGGTC

GGTACAGAGGGTTGCCAACCCGCGAGGGGGAGCCAATCCCAGAAAGCCGA

TCGTAGTCCGGATTGTAGTCTGCAACTCGACTACATGAAGTCGGAATCGC

TAGTAATCGCGGATCAGCATGTCGCGGTGAATACGTTCCCGGGTCTTGTA

CACACCGC

>S10_18410|HM243953

AGAGTTTGATCCTGGCTCAGAACGAACGCTGGCGGCGTGCCTAACACATG

CAAGTCGTACGAGATAGCCCCTTCGGGGGTGTGTAAAGTGGCGCACGGGT

GAGTAACGCGTAGATAATCTGCCCTGGTATCTGGGATAACATCTCGAAAG

GGGTGCTAATACCGGATAAGCCCACGACGGCTTTGGTCGTTGCGGGAAAA

GGGGGGGACCTTCGGGCCTACCGTATCAGGATGGGTCCGCGTACCATTAG

CTAGTTGGTGGGGTAATGGCCTACCAAGGCGACGATGGTTAGCTGGTCTG

AGAGGATGATCAGCCACACTGGAACTGAGACACGGTCCAGACTCCTACGG

GAGGCAGCAGTGGGGAATTTTGCGCAATGGGGGAAACCCTGACGCAGCAA

CGCCGCGTGAGTGATGAAGGCTTTCGGGTCGTAAAGCTCTGTCAGAGGGG

AAGAAATGGGTCGTGCTAATATCGCGACTTCTTGACGGTACCCTCAAAGG

AAGCACCGGCTAACTCCGTGCCAGCAGCCGCGGTAATACGGAGGGTGCAA

GCGTTGTTCGGATTTATTGGGCGTAAAGCGCGTGTAGGCGGTTTTTTAAG

TCTGATGTGAAAGCCCTGGGCTCAACCCAGGAAGTGCATTGGATACTGGA

AGACTTGAATACGGGAGAGGGTAGTGGAATTCCTAGTGTAGGAGTGAAAT

CCGTAGATATTAGGAGGAACACCGGTGGCGAAGGCGGCTACCTGGACCGA

TATTGACGCTGAGACGCGAAAGCGTGGGGAGCAAACAGGATTAGATACCC

TGGTAGTCCACGCCGTAAACGATGAGAACTAGGTGTTGCGGGTATTGACC

CCTGCAGTGCCGCAGCTAACGCATTAAGTTCTCCGCCTGGGAAGTACGGT

CGCAAGACTAAAACTCAAAGGAATTGACGGGGGCCCGCACAAGCGGTGGA

GCATGTGGTTTAATTCGACGCAACGCGCAGAACCTTACCTGGGCTTGACA

TCTGCGGAATCTTCGTGAAAGCGAGGAGTGCCTTCGGGAGCCGCAAGACA

GGTGCTGCATGGCTGTCGTCAGCTCGTGTCGTGAGATGTTGGGTTAAGTC

CCGCAACGAGCGCAACCCCTATCCTTAGTTGCCATCATTCAGTTGGGCAC

TCTAAGGAGACTGCCGGTGTCAAACCGGAGGAAGGTGGGGATGACGTCAA

GTCCTCATGGCCCTTATGTCCAGGGCTACACACGTGCTACAATGGCCGGT

ACAAAGGGTTGCGATACCGCGAGGTGGAGCCAATCCCAAAAAGCCGGTCT

CAGTTCGGATTGGAGTCTGCAACCCGACTCCATAAAGTCGGAATCGCTAG

TAATCGCGGATCAGCATGCCGCGGTGAATACGTTCCCGGGCCTTGTACAC

ACCGCCCGTCACACCATGGGAGTCGATTGGTCCCGAAGTGCGTGAGCTAA

CCCGCAAGGGAAGCAGCGTCCTACGGACTGGTCGGTGACTGGGGTGAAGT

CGTAACAAGGTAACC

>S10_17729|EU193072

AGACAGGGATAACATCTCGAAAGGGGTGCTAATACCGGATAAGCCTACGG

ACTCTTCGGAGTCTGCAGGAAAAGGTGGCCTCTATTTATAAGCTACCGTA

TCAGGATGAGTCTGCGTACCATTAGCTAGTTGGTAGGGTAATGGCCTACC

AAGGCTACGATGGTTAGCTGGTCTGAGAGGATGATCAGCCACACTGGAAC

TGAGACACGGTCCAGACTCCTACGGGAGGCAGCAGTGGGGAATTTTGCGC

AATGGGCGAAAGCCTGACGCAGCAACGCCGCGTGAGTGATGAAGGCTTTC

GGGTCGTAAAGCTCTGTCGAGGGGAAAGAAATGTACGAGGGCTAATATCC

TTTGTACTTGACGGTACCCCTAAAGGAAGCACCGGCTAACTCCGTGCCAG

CAGCCGCGGTAATACGGGGGGTGCAAGCGTTGTTCGGAATTATTGGGCGT

AAAGCGCGTGTAGGCGGTTTGTTAAGTCTGATGTGAAAGCCCTGGGCTCA

ACCCAGGAAGTGCATTGGAAACTGGCAGACTTGAATACGGGAGAGGGTAG

TGGAATTCCTAGTGTAGGAGTGAAATCCGTAGATATTAGGAGGAACACCG

GTGGCGAAGGCGGCTACCTGGACCGATATTGACGCTGAGACGCGAAAGCG

TGGGTAGCAAACAGGATTAGATACCCTGGTAGTCCACGCCGTAAACGATG

AGTACTAGGTGTTGCGGGTATTGACCCCTGCAGTGCCGCAGCTACGCATT

AAGTACTCCGCCTGGGAAGTACGGTCGCAAGACTAAAACTCAAAGGAATT

GACGGGGGCCCGCACAAGCGGTGGAGCATGTGGTTTAATTCGACGCAACG

CGCAGAACCTTACCTGGGCTTGACATCTGCGGAACCTCTGTGAAAGCAGG

GGGTGCCCGAAAGGGAGCCGCAAGACAGGTGCTGCATGGCTGTCGTCAGC

TCGTGTCGTGAGATGTTGGGTTAAGTCCCGCAACGAGCGCAACCCCTACC

CTTAGTTGCCAGCATTAAGTTGGGCACTCTATGGGGACTGCCGCAGACAA

TGTGGAGGAAGGTGGGGATGACGTCAAGTCCTCATGGCCTTTATGCCTAG

GGCTACACACGTACTACAATGGCCGTTACAGAGGGCAGCAAGCTCGCGAG

GGTAAGCAAATCCCAGAAAGACGGCCTCAGTTCGGATTGGAGTCTGCAAC

TCGACTCCATGAAGTCGGAATTGCTAGTAATCGCTGATCAGCAGGCAGCG

GTGAATACGTTCCCGGGCCTTGTACACACCGCCCGTCACACCACGAAAGT

CTGTTATACCCGAAGTCGGTGGGCTAACCGCAAGGGGGCAACTGCCTAAG

GTATGGCCGATGATTGGGGTGAAGTCGTAACAAGGTAACC
