# Supplementary material for: Fluctuations in Species-Level Protein Expression Occur during Element and Nutrient Cycling in the Subsurface
Source: PLoS One. 2013 Mar 5;8(3):e57819. doi: 10.1371/journal.pone.0057819 (PMC3589452; doi:10.1371/journal.pone.0057819)
Supplement: Table S2 — Nucleotide % similarity between full length 16S rRNA sequences from environmental and sequenced strains. (DOCX) [file pone.0057819.s004.docx]

| Clones | Gbem | GM21 | GM18 |
| --- | --- | --- | --- |
| GW1_266\|CP001124 | 97.27 | 97.76 | 96.97 |
| GW2_4\|CP001124 | 97.49 | 97.89 | 97.17 |
| GW2_5951\|DQ133931 | 96.3 | 95.21 | 94.62 |
| GW2_1044\|FJ902092 | 95.75 | 95.59 | 95.43 |
| GW2_8636\|CP002431 | 94.75 | 93.7 | 92.71 |
| GW3_4\|CP001124 | 97.49 | 97.89 | 97.17 |
| GW3_8153\|JF344521 | 94.08 | 94.02 | 93.41 |
| GW3_3844\|FJ810552 | 93.78 | 92.66 | 91.93 |
| GW3_3844\|FJ810552 | 95.24 | 96.12 | 96.15 |
| GW3_476\|HQ183850 | 95.39 | 93.68 | 93.11 |
| GW3_14238\|GQ500708 | 94.97 | 93.45 | 93.71 |
| GW3_15078\|GQ500708 | 94.03 | 92.89 | 92.88 |
| GW3_15346\|HM748811 | 95.08 | 95.46 | 95.34 |
| GW4_9\|CP001124 | 97.35 | 97.63 | 97.03 |
| GW4_18296\|FR667779 | 93.91 | 94.61 | 93.9 |
| GW4_18109\|FJ793176 | 92.6 | 93.56 | 93.18 |
| GW4_20170\|JF736631 | 94.92 | 95.78 | 94.93 |
| GW4_20605\|EU236221 | 95.16 | 95.63 | 95.49 |
| GW4_936\|EU244081 | 93.34 | 93.6 | 93.32 |
| GW4_18161\|AY780557 | 94.07 | 94.55 | 95.15 |
| GW5_51\|CP001124 | 97.35 | 97.63 | 97.03 |
| GW5_71\|FR667782 | 92.54 | 93.19 | 93.13 |
| GW5_475\|AY013648 | 91.45 | 91.54 | 91.45 |
| GW5_18410\|HM243953 | 94.93 | 95.13 | 94.21 |
| GW5_17729\|EU193072 | 93.78 | 93.08 | 92.5 |
