# Supplementary material for: Fluctuations in Species-Level Protein Expression Occur during Element and Nutrient Cycling in the Subsurface
Source: PLoS One. 2013 Mar 5;8(3):e57819. doi: 10.1371/journal.pone.0057819 (PMC3589452; doi:10.1371/journal.pone.0057819)
Supplement: Table S5 — Proteins exhibiting significant changes in abundance between the three stages of biostimulation, displayed as a percentage of the total number of proteins detected across the experiment (925). (DOCX) [file pone.0057819.s007.docx]

|  |  | Early > Mid | Mid > Early | Late > Mid | Mid > Late |
| --- | --- | --- | --- | --- | --- |
| Translation, ribosomal structure and biogenesis | J | 2.9 | 2.1 | 0.0 | 10.2 |
| RNA processing and modification | A | 0.0 | 0.0 | 0.0 | 0.0 |
| Transcription | K | 1.1 | 0.8 | 0.0 | 4.2 |
| Replication, recombination and repair | L | 0.3 | 0.9 | 0.0 | 2.4 |
| Chromatin structure and dynamics | B | 0.0 | 0.0 | 0.0 | 0.0 |
| Cell cycle control, cell division, chromosome partitioning | D | 0.1 | 0.2 | 0.0 | 0.9 |
| Nuclear structure | Y | 0.0 | 0.0 | 0.0 | 0.0 |
| Defense mechanisms | V | 0.1 | 0.0 | 0.0 | 0.2 |
| Signal transduction mechanisms | T | 2.1 | 2.2 | 0.0 | 10.7 |
| Cell wall/membrane/envelope biogenesis | M | 0.6 | 1.5 | 0.0 | 5.5 |
| Cell motility | N | 1.1 | 1.6 | 0.0 | 7.4 |
| Cytoskeleton | Z | 0.0 | 0.0 | 0.0 | 0.0 |
| Extracellular structures | W | 0.0 | 0.0 | 0.0 | 0.0 |
| Intracellular trafficking, secretion, and vesicular transport | U | 0.5 | 0.9 | 0.0 | 3.7 |
| Posttranslational modification, protein turnover, chaperones | O | 0.5 | 1.3 | 0.0 | 3.8 |
| Energy production and conversion | C | 1.1 | 3.9 | 0.0 | 9.7 |
| Carbohydrate transport and metabolism | G | 0.3 | 0.9 | 0.0 | 3.7 |
| Amino acid transport and metabolism | E | 1.1 | 2.2 | 0.0 | 10.3 |
| Nucleotide transport and metabolism | F | 0.5 | 0.6 | 0.0 | 3.2 |
| Coenzyme transport and metabolism | H | 0.8 | 0.6 | 0.0 | 4.6 |
| Lipid transport and metabolism | I | 0.3 | 0.6 | 0.0 | 2.6 |
| Inorganic ion transport and metabolism | P | 0.6 | 1.1 | 0.0 | 2.1 |
| Secondary metabolites biosynthesis, transport and catabolism | Q | 0.0 | 0.1 | 0.0 | 1.1 |
| General function prediction only | R | 0.5 | 1.7 | 0.0 | 5.9 |
| Function unknown | S | 0.1 | 1.2 | 0.0 | 3.9 |
